# Decoding the Mechanism of Action of a Parasite TGFβ Antagonist Inspires the Creation of Cell‐Type‐Specific TGFβ Modulators

**DOI:** 10.1002/advs.75322

**Published:** 2026-04-20

**Authors:** Maarten van Dinther, Tristin Schwartze, Jiying Zhang, Kun Fan, Gerard van der Zon, Luke Power, Cynthia S. Hinck, Claire Ciancia, Ananya Mukundan, Roman Gonzalez‐Prieto, Peter van Veelen, Rick M. Maizels, Andrew P. Hinck, Peter ten Dijke

**Affiliations:** ^1^ Oncode Institute and Department of Cell and Chemical Biology Leiden University Medical Center Leiden The Netherlands; ^2^ Department of Structural Biology University of Pittsburgh School of Medicine Pittsburgh Pennsylvania USA; ^3^ Centre for Parasitology School of Infection and Immunity University of Glasgow Glasgow UK; ^4^ Andalusian Center for Molecular Biology and Regenerative Medicine Universidad de Sevilla ‐ CSIC ‐ Universidad Pablo de Olavide Sevilla Spain; ^5^ Department of Cell Biology Faculty of Biology University of Sevilla Sevilla Spain; ^6^ Center for Proteomics and Metabolomics Leiden University Medical Center Leiden The Netherlands

**Keywords:** betaglycan, bispecific antibodies, heligmosomoides polygyrus, lipoprotein receptor‐related protein, molecular mimicry, signal transduction, transforming growth factor‐β

## Abstract

*Heligmosomoides polygyrus*, a mouse parasite, modulates host immunity by secreting modular transforming growth factor‐β (TGFβ) mimics (TGMs). The agonist TGM1 interacts with TGFBR1, TGFBR2, and the co‐receptor CD44 through domains D1/2, D3, and D4/5, respectively. In contrast, the antagonist TGM6, which lacks D1/2, but retains TGFBR2 binding through D3, targets different cells compared to TGM1. The TGM6 co‐receptor is unknown. Using X‐ray crystallography and binding studies, we show that TGM6 preferentially binds mouse TGFBR2 over human TGFBR2, and that this is essential for its antagonistic function. We identified low‐density lipoprotein receptor‐related protein 1 (LRP1) and betaglycan (TGFBR3) as co‐receptors for TGM6. LRP1 enhances TGM6 efficacy and is required for its antagonistic effect by promoting TGFBR2 lysosomal degradation, whereas betaglycan counteracts TGM6 in a TGFBR2‐dependent manner. The modular organization of TGMs enabled us to design TGM1/6 chimeras or TGM‐D3 fusion with an affibody that recognizes a specific cell‐surface receptor, thereby altering cell‐type specificity and functionality. Furthermore, we developed a TGFBR2 nanobody that, on its own, has no inhibitory effect but, when fused to a receptor antibody, antagonizes TGFβ by blocking TGFβ receptor interaction in a cell‐selective manner. Thus, we designed programmable agents that modulate TGFβ signaling only in co‐receptor‐expressing cells.

## Graphical abstract

The mammalian TGFβ interacts with ubiquitously expressed TGFBR1 and TGFBR2, and current TGFβ‐targeting agents are non‐cell‐selective. The cooperative interaction of the modular parasite TGFβ antagonist with multiple host (co‐)receptors empowers the design of TGM chimeras and bispecific antibodies that activate or inhibit TGFβ signaling in a cell‐selective manner.

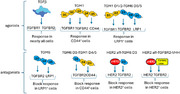

## 1 Introduction

Transforming growth factor‐β (TGFβ) is a secreted cytokine that exerts many diverse cellular responses via specific cell surface heteromeric complexes of two ubiquitously expressed TGFβ receptors, i.e., TGFBR1 and TGFBR2 [1, 2, 3]. TGFBR1 and TGFBR2 are essential for TGFβ signaling [4]. TGFBR1 acts downstream of TGFBR2 [4], and when activated, TGFBR1 induces the phosphorylation of receptor‐regulated (R) SMAD2 and SMAD3 proteins [1, 2, 3]. Upon activation, R‐SMADs form heteromeric complexes with SMAD4, which accumulate in the nucleus and regulate specific gene transcriptional responses [5].

TGF‐β plays a pivotal role in physiological processes, including embryonic development, wound healing, and tissue remodeling, as well as in maintaining immune tolerance and preventing autoimmunity. Dysregulated, overactive TGFβ signaling can contribute to pathological responses, such as fibrosis and cancer [1]. However, it is often unclear which critical cell types the multifunctional TGF‐β acts on in complex physiological and pathological processes. The same holds for results from studies using current TGFβ inhibitors, such as TGFβ‐neutralizing antibodies, TGFBR2‐extracellular domain (ECD)‐derived ligand traps, and small‐molecule TGFBR1 kinase inhibitors, which inhibit TGFβ responses across all cell types [6]. For example, the underlying mechanism by which the TGFβ antagonist inhibits cancer progression is often unclear: does it antagonize cancer cell invasion, inhibit activation of cancer‐associated fibroblasts, or inhibit immune evasion and stimulate immune cell infiltration into tumors? Moreover, the lack of cell specificity of current TGFβ inhibitors limits their clinical translation. Because the inhibitors also interfere with the TGFβ's normal homeostatic functions, they may induce serious side effects, including cardiac toxicity and skin tumors [6]. As a result, TGFβ inhibitors have not yet been approved for the treatment of cancer or fibrosis.

The murine helminth parasite *Heligmosomoides polygyrus* (Hp) modulates host immune responses by secreting TGFβ mimics (TGMs) [7]. The prototypic TGM1 exerts TGF‐β‐like effects on immune cells in vitro [8, 9, 10] and in vivo [8, 10, 11, 12, 13, 14, 15]. In addition to TGM1, 9 different structurally related HpTGMs have been identified [16]. HpTGMs have no primary sequence similarity to mammalian TGFβ. In contrast to the compact globular protein structure of mammalian TGFβ, TGMs have a modular structure with individual domains that are distantly related to the Sushi domain family or to complement control proteins (CCPs) [17]. Its prototypic member, TGM1, phenocopies the immunosuppressive effects of TGFβ by stimulating T regulatory cells [8]. TGM1 has five separate domains (D) in which D1/2, D3, and D4/5 interact with TGFBR1, TGFBR2, and co‐receptor CD44, respectively [17, 18]. CD44‐deficient T regulatory cells exhibited impaired TGM1‐mediated induction of the transcription factor FoxP3 [18]. TGM6 lacks D1/2 and, therefore, TGFBR1 engagement, but retains TGFBR2 interaction via its D3. TGM6 is a potent antagonist of TGFBR signaling in fibroblasts, but not splenic T cells [19]. D4/5 of TGM6 is essential for antagonism of TGFβ, but does not bind CD44 [19]. D4/5 is the most divergent among the TGM domains [16], and emerging studies indicate that it is a key determinant of the cell‐specific effects elicited by different TGMs [18, 19, 20].

TGM6 is secreted by a parasite that infects mice, not humans, and has evolved through convergent evolution [21]. Here, we report the molecular mechanisms underlying the selective action of TGM6 on mouse but not human cells. Importantly, we identified low‐density lipoprotein receptor‐related 1 (LRP1) and betaglycan (TGFBR3) as two opposing TGM6 co‐receptors. Moreover, the modular organization of TGMs enabled us to rationally design TGM1/6 chimeras, thereby altering the cell‐type specificity of TGM6 and transforming the TGM6 antagonist into a mouse‐cell‐type‐selective agonist or antagonist. Furthermore, we developed a TGFBR2 nanobody, which, when fused to a receptor antibody, antagonizes TGFβ signaling in a human cell‐selective manner. Our identified principles may be instrumental in developing therapies targeting the highly pleiotropic TGFβ in patients with dysregulated TGFβ signaling.

## 2 Results

### 2.1 TGM6‐Mediated Inhibition of TGFβ Signaling Is Effective on Mouse and Rat, but not Human Cells

**FIGURE 1:**
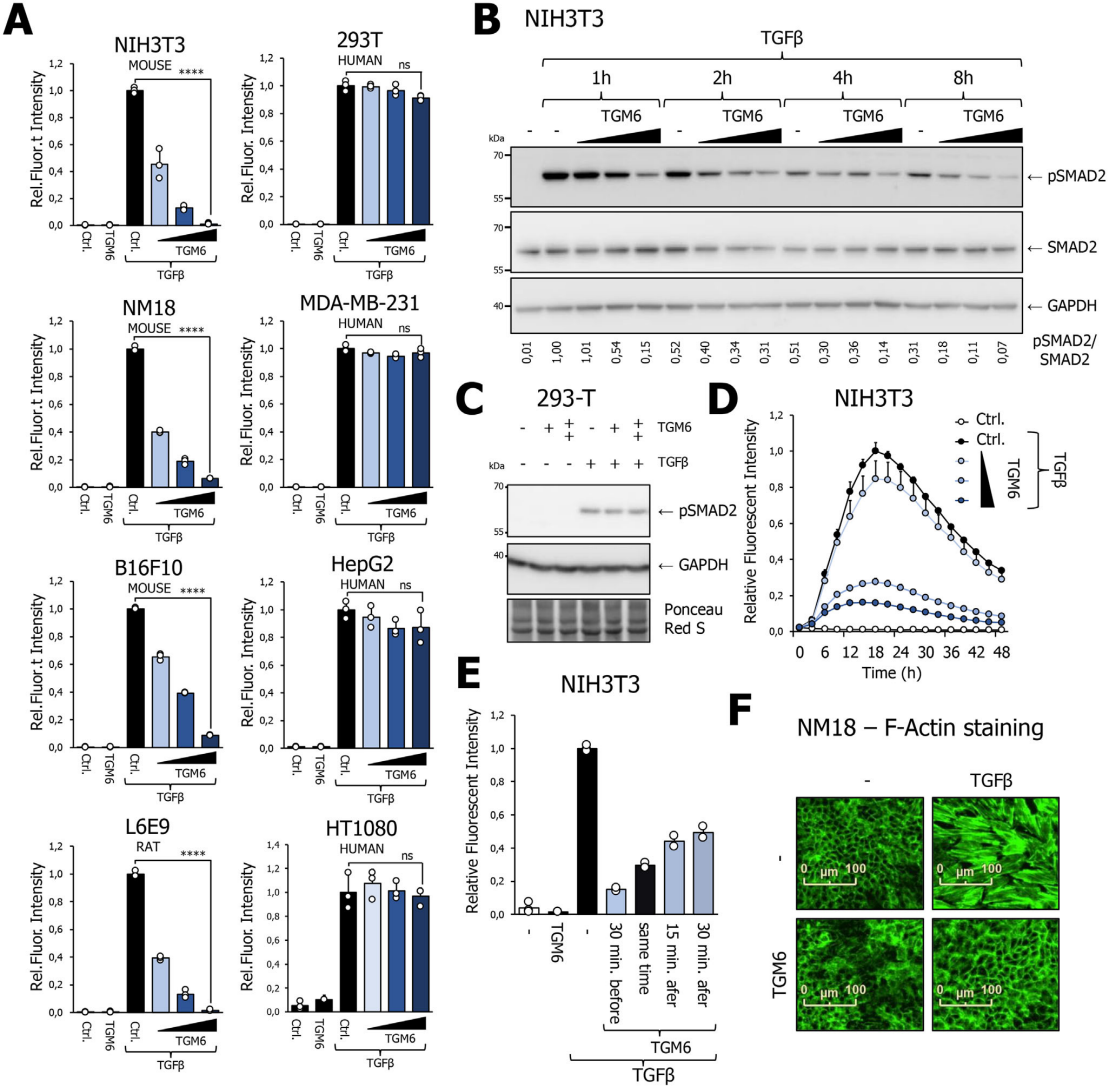
TGM6 potently antagonizes TGFβ/SMAD signaling in mouse, rat, but not human cells. (A) Effect of TGM6 on TGFβ/SMAD3‐induced transcriptional response in mouse, rat, and human cells. Mouse NIH3T3 fibroblasts, NMuMG breast cells (NM18 clone), B16F10 melanoma, rat L6E9 myoblasts and human 293T epithelial, MDA‐MD‐231 breast cancer, HepG2 hepatoma and HT1080 fibrosarcoma cells that were engineered to express a CAGA‐dynGFP reporter were pre‐treated for 30 min with different doses of TGM6 (10, 25, or 100 ng/ml) and were subsequently stimulated with TGFβ for 24 h. (B, C) Effect of TGM6 on TGFβ‐induced SMAD2 phosphorylation in NIH3T3 cells and HEK293T cells. (B) NIH3T3 cells were pre‐treated for 30 min with TGM6 (10, 25, or 100 ng/ml) and subsequently stimulated with TGFβ for 1, 2, 4 or 8 h. The pSMAD2/SMAD2 ratios are indicated below the GAPDH blot results in B. (C) HEK293T cells were pre‐incubated with 100 ng/ml TGM6 and subsequently treated with TGFβ. Cells were also treated with TGFβ alone. (D) Kinetic effect of TGM6 on NIH3T3 CAGA‐dynGFP reporter activity; cells were treated with TGFβ and different doses of TGM6 (10, 25, or 100 ng/ml), (E) Effect of different exposure times to TGM6 on the inhibitory effect on TGFβ/SMAD signaling: NIH3T3 cells containing the CAGA‐dynGFP reporter were pre‐treated, treated at the same time, or treated with 100 ng/ml TGM6 and TGFβ (21 h). (F) Effect of TGM6 100 ng/ml on TGFβ‐induced EMT in mouse NM18 breast cells. The TGFβ concentration used in all the aboive experiments was 1 ng/ml.

TGM6 mimics the interaction of TGFβ with TGFBR2, and antagonizes TGFβ signaling in specific cells, including fibroblasts, but not other cell types, such as T cells [19], in a manner dependent on two C‐terminal co‐receptor‐binding domains, D4/5. Although TGM6 D4/5 differs from TGM1 D/5 in that it does not bind CD44 [19], the identity of TGM6 co‐receptors remains unknown. In our survey of TGM6‐responsive cell lines to select appropriate cells for TGM6 co‐receptor identification and mechanism‐based studies, we found that TGM6 potently blocked the TGFβ/SMAD signaling response in mouse and rat cells, but not in human cells (Figure 1A). In none of the cells did we observe TGFβ agonistic activity by TGM6 alone (Figure 1A). TGM6 was highly effective in blocking TGFβ‐induced SMAD3/4 transcriptional response in mouse NIH3T3 fibroblasts (Figure 1A), and these cells were chosen for further investigation. Analyzing the kinetics of TGM6 inhibition on TGFβ in NIH3T3 cells showed that TGM6 could mitigate TGFβ/SMAD2 phosphorylation after 1 h to 8 h stimulation time (Figure 1B). Consistent with the lack of an effect of TGM6 on TGFβ‐induced transcriptional response in human 293T cells, TGM6 did not affect TGFβ‐induced SMAD2 phosphorylation (Figure 1C). Increasing TGM6 doses effectively inhibited the TGFβ‐induced SMAD3/4 transcriptional response in NIH3T3 cells (Figure 1D). Adding TGM6 before TGFβ was more efficient than adding it simultaneously or shortly thereafter (Figure 1E). TGFβ is known for being a potent inducer of epithelial‐to‐mesenchymal transition (EMT) [22]. TGM6 blocked TGFβ‐induced EMT of mouse NMuMG breast cells (Figure 1F). Taken together, our results indicate that TGM6 is a potent antagonist of TGFβ in rodent, but not human, cells.

### 2.2 Three Amino Acid Differences Between the Mouse and Human TGFBR2 Extracellular Domains Are Major Determinants for TGM6 Species Specificity

**FIGURE 2:**
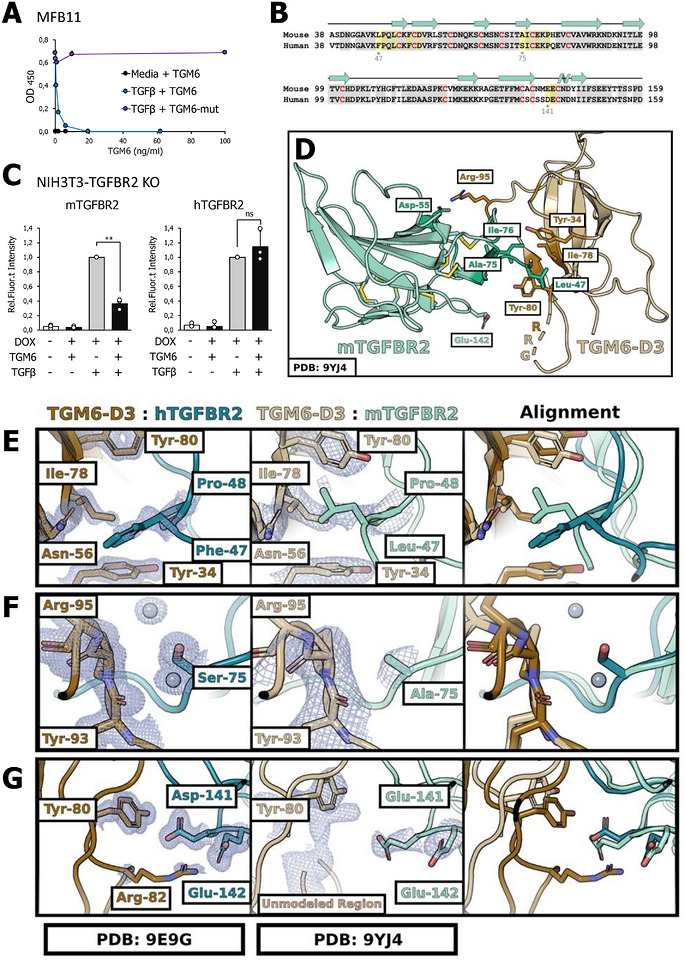
Three amino acid differences in the mouse and human TGFBR2 extracellular domain are responsible for TGM6 species specificity. (A) Effect of TGM6 (wildtype) or TGM6‐mut (defective in TGFBR2 interaction) on TGFβ (5 ng/ml)‐induced activation of SMAD3 transcriptional activity using MFB‐F11 reporter cells. (B) Amino acid sequence alignment of extracellular domains of mTGFBR2 and hTGFBR2. Identical non‐interfacial and interfacial residues between mouse and human are highlighted with grey or dark yellow shading, respectively. Non‐identical residues are not shaded. The three residues at the interface that are expected to differ between the complexes with hTGFBR2 (Phe47, Ser75, Asp141) and mTGRBR2 (Leu47, Ala 5, Glu141) are highlighted in light yellow with an asterisk. Conserved cysteine residues are indicated in red, and the β strands and α−helix are indicated on top of the primary amino acid sequence with blue arrows and blue helix, respectively. (C) Mouse, but not human, TGFBR2 can restore TGM6‐induced antagonism of TGFβ/SMAD signaling in TGFBR2‐knockout cells. NIH3T3 TGFBR2 knock‐out cells were transduced with lentiviruses containing either mouse or human TGFBR2 under a doxycycline‐inducible promoter. TGFBR2 expression was induced overnight by doxycycline, and cells were pretreated with 100 ng/ml TGM6 before stimulation with 1 ng/ml TGFβ; samples were analyzed by Western blotting for SMAD2 phosphorylation. The integrated results from three independent experiments are shown. (D) Overall structure of the mTGFBR2:TGM6‐D3 ECD complex determined by X‐ray crystallography at a resolution of 2.52 Å. mTGFBR2 ECD and TGM6‐D3 are shaded teal and light brown, respectively. Loops that are not modeled due to weak density are indicated by dashed lines. Side chains of key interfacial residues, including those that differ relative to those in hTGFBR2, are shown, as are the intramolecular disulfide bonds (E‐G). Comparison of TGM6‐D3 in complex with human or mouse TGFBR2 at comparable interface regions at positions that differ between human and mouse TGFBR2. The left and middle panels show the model density for the human and mouse complexes, respectively, while the right panel shows an alignment of the human and mouse models. In (E), Leu47 in mTGFBR2 is shown to fill the pocket formed by TGM6‐D3 Tyr34, Ile78, and Tyr80 – in hTGFBR2, Phe47 in is unable to rotate inward to fill the pocket—hence this interface difference likely contributes to the preferential binding of mTGFBR2 to TGM6‐D3. In (F), Ala75 in mTGFBR2 is shown to adopt essentially the same position as Ser^75^ in hTGFBR2, and so may only make a minor contribution to the preferential binding of mTGFBR2 to TGM6‐D3. In (G), both Glu142 in mTGFBR2 and Asp142 in hTGFBR2 are positioned close to a highly mobile loop in TGM6‐D3, and this interface difference might also only contribute to the preferential binding of mTGFBR2 to TGM6‐D3 in a minor way.

TGM6 binds TGFBR2 with high affinity [19]. To investigate the functional importance of this interaction, we mutated three TGM6 amino acid residues, i.e., Arg38, Ile78, and Tyr93 to Ala residues in TGM6 (TGM6‐mut), which are critical for TGFBR2 interaction based upon TGM6:hTGFBR2 extracellular domain (ECD) crystal structure [19], and analysed the effect of TGM6‐mut on TGFβ/SMAD signaling in mouse MFB‐F11 fibroblasts. TGM6‐mut was fully inactive, suggesting that TGM6‐TGFBR2 interaction is essential for the antagonistic function of TGM6 (Figure 2A). When we compared the mouse (m) and human (h) TGFBR2 ECDs, we found 20/122 amino acid differences in the extracellular ligand binding domain, including 3 in the previously identified interface between TGM6‐D3 and hTGFBR2 (Figure 2B). We therefore examined whether this dissimilarity is a crucial determinant of differential TGM6 activity. We ectopically expressed mTGFBR2 and hTGFBR2 (in a doxycycline‐inducible manner) in NIH3T3 cells, which were deficient in mTGFBR2 due to CRISPR‐CAS9 gene editing, and subjected the cells to TGFβ in the absence or presence of TGM6 and analyzed phosphorylated SMAD2 levels. We found that mTGFBR2, but not hTGFBR2, restored TGM6 responsiveness (Figure 2C), suggesting that mTGFBR2 is a critical mediator of TGM6 antagonist activity.

To further investigate whether the increased antagonistic activity of TGM6 in mouse cells is due to preferential binding to mTGFBR2, we measured the binding affinity of purified TGM6‐D3 for purified mTGFBR2 and hTGFBR2 using isothermal titration calorimetry (ITC) (Figure S1). These binding measurements, which were performed with duplicate titrations and global analysis of the integrated heats [23, 24], showed that TGM6‐D3 bound mTGFBR2 with 40‐fold greater affinity compared to hTGFBR2 (15 nM vs. 604 nM, Table 1).

**TABLE 1:** TGM6:TGFBR2 binding as assessed by ITC.

	hTGFBR2‐WT	hTGFBR2‐F47L	hTGFBR2‐3M	mTGFBR2‐WT	mTGFBR2‐L47F	mTGFBR2‐3M
[Cell, TGM6‐D3] (microµM)	10	10	10	10	10	10
[Syr, TGFBR2] (microµM)	85	85	115	90	90	90
Cell Incomp (%)^a^, ^b^	2 (0,6)	0 (0,4)	0 (0,4)	0 (0,3)	0 (0,7)	0 (0,39)
Syr Incomp (%)^a^, ^b^	1 (0,4)	16 (15,19)	41 (41,43)	8 (7, 11)	22 (20,26)	9 (7,12)
K_D_ (nM)^a^, ^b^	604 (794,456)	99 (138,69)	38 (52,27)	15 (24,9)	165 (259,101)	655 (908,472)
∆H (kcal mol^−1^)^a^, ^b^	−16.7 (−17.9,−15.7)	−18.9 (−20.0,−17.9)	−18.2 (−19.0, −17.4)	−21.8 (−22.7, −20.9)	−17.6 (−19.3, −16.0)	−19.5 (−21.1,−18.0)
∆G (kcal mol^−1^)	−8.8	−9.9	10.5	−11.0	−9.6	−8.7
‐T∆S (kcal mol^−1^)	8.0	9.0	7.7	10.8	8.0	10.8
T (°C)	35	35	35	35	35	35

^a^ Derived from global fit of two independent titrations

^b^ Uncertainty reported as ± 1𝜎.

The structure of the TGM6‐D3:hTGFBR2 ECD complex [19] shows that only three interface residues are expected to differ between the complex with hTGFBR2 (Phe47, Ser75, Asp141) and mTGRBR2 (Leu47, Ala75, Glu141). Thus, we hypothesized that these three residues are responsible for the strong preferential binding of TGM6‐D3 to mTGFBR2. To investigate this, we crystallized the TGM6‐D3:mTGFBR2 complex and determined its structure at 2.52 Å resolution by X‐ray crystallography (Table S1). This showed that the overall structure of the TGM6‐D3:mTGFBR2 complex was very similar to the previously reported structure of the TGM6‐D3:hTGFBR2 complex, with an overall root mean square deviation (RMSD) over all heavy atoms of 0.49 ‐ 0.58 Å (Figure 2D, S2). The close similarity was unsurprising, given that mouse and human TGFBR2 are 83% identical across the entire ECD.

Through closer inspection of the structures of TGM6‐D3 in complex with mTGFBR2 and hTGFBR2, we found that the side chains of the conserved interface residues engage TGM6‐D3 in an essentially identical manner. There were nonetheless significant differences for one of the non‐conserved interface residues, residue 47 (Figure 2E). In the structure of the mouse complex, Leu47 adopts a sidechain rotamer conformation that allows it to fill a hydrophobic pocket formed by TGM6‐D3 Tyr34, Ile78, and Tyr80. In the human complex, Phe47 could enter the pocket only in a high‐energy conformation due to its reduced conformational flexibility relative to leucine. In contrast to residue 47, only minor structural differences and no apparent changes in interactions are observed for residues 75 and 141 that might account for the preferential binding of TGM6‐D3 for mTGFBR2 (Figure 2F,G).

To test our hypothesis that the preferential binding of TGM6‐D3 for mTGFBR2 is engendered primarily by Leu47 (in place of Phe47 in hTGFBR2), we expressed and purified the hTGFBR2 F47L and mTGFBR2 L47F single amino acid variants and hTGFBR2 F47L, S75A, D141E and mTGFBR2 L47F, A75S, and E141D triple amino acid variants (hTGFBR2‐3M and mTGFBR2‐3M, respectively) and measured their binding affinity for TGM6‐D3 using ITC. These binding measurements, which were performed with duplicate titrations and global analysis of the integrated heats [23, 24], showed that the three residues were indeed responsible for the preferential binding, with the hTGFBR2‐3M gain‐of‐function and the mTGFBR2‐3M loss‐of‐function variants binding comparably to mTGFBR2‐WT and hTGFBR2‐WT, respectively (Table 1). The data further showed that residue 47 is the primary determinant of preferential binding, as the hTGFBR2‐F47L and mTGFBR2‐L47F variants gained and lost nearly two‐thirds of the binding affinity relative to mTGFBR2‐WT and hTGFBR2‐WT, respectively (Table 1). Together, these data show that TGM6 preferentially antagonizes signaling in mouse cells due to a 30‐40‐fold preference for binding mTGFBR2 over hTGFBR2.

### 2.3 TGM6 Binds to TGFBR2 and Betaglycan and Competes With TGFβ for TGFBR2 Binding

**FIGURE 3:**
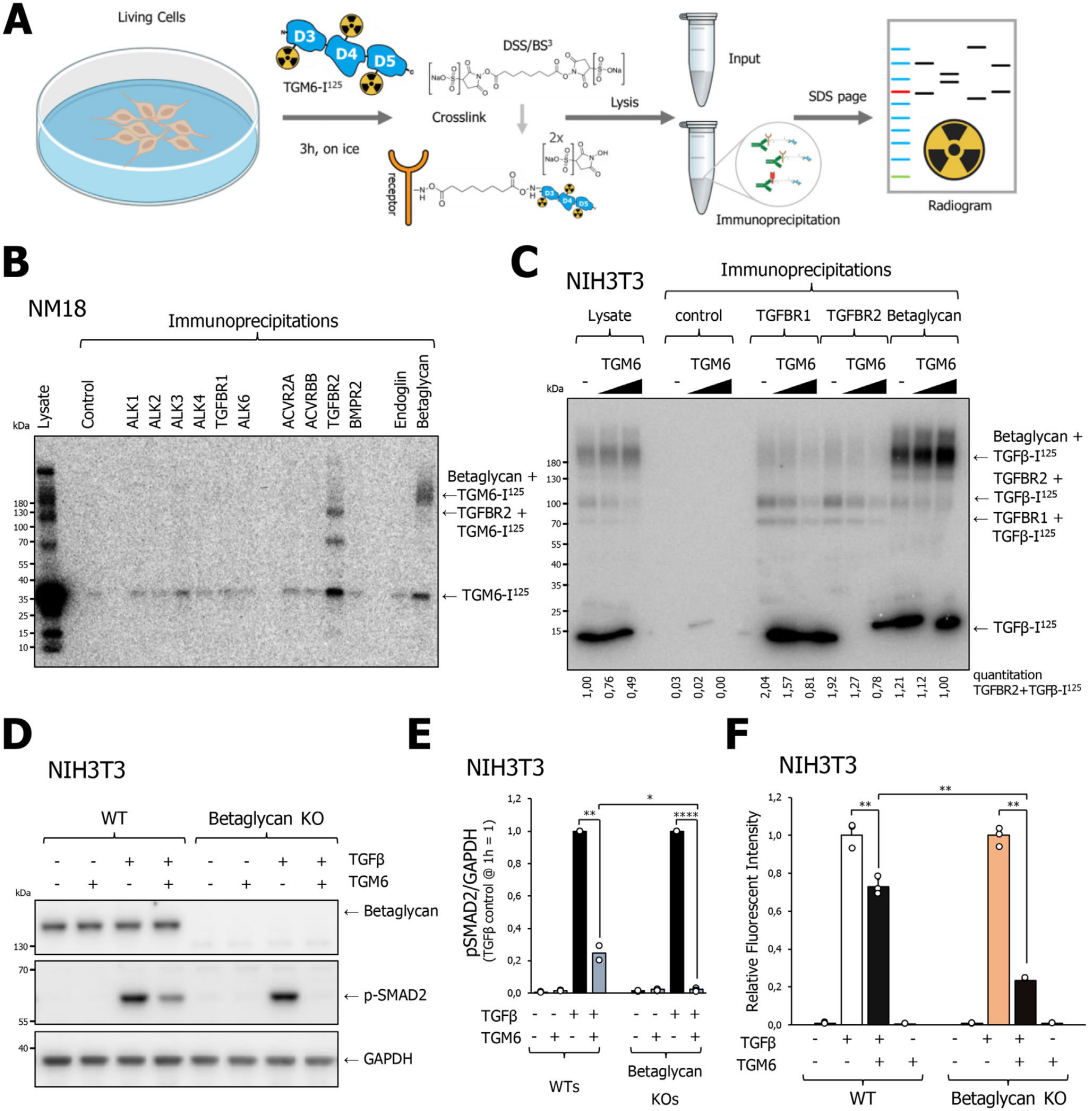
TGM6 competes with TGFβ for TGFBR2 interaction, and binds Betaglycan, a negative regulator of TGM6 function.(A) Schematic of the experimental flow of the affinity labeling experiment of NIH3T3 cells with iodinated TGM6. (B) Analysing the binding of TGM6 to TGFβ family type I, type II, and type III receptors. NM18 cells were incubated with iodinated TGM6, then crosslinked, lysed, and incubated overnight with receptor‐specific antibodies. Thereafter, complexes were isolated using protein A beads, separated by SDS‐PAGE, and detected by autoradiography. (C) Effect of TGM6 on radiolabeled TGFβ binding to TGFBR1, TGFBR2, and betaglycan. NIH3T3 cells were pre‐treated with TGM6 for 30 min, then incubated with iodinated TGFβ, crosslinked, lysed, and incubated overnight with receptor‐specific antibodies; finally, complexes were isolated using protein A beads, separated by SDS‐PAGE, and detected by autoradiography. The relative intensity of TGFBR2‐TGFβ‐I^125^ is indicated below the autoradiogram. (D and E) Western blot analysis for the TGM6 effect on TGFβ‐induced SMAD2 phosphorylation in wild‐type NIH3T3 cells or derivatives deficient in betaglycan. (D) Representative pSMAD2 Western blotting results. (E) Integration and quantification of pSMAD2 to GAPDH expression ratios of multiple wild‐type or knockout clones. (F) Effect of betaglycan deficiency on the ability of TGM6 to antagonize TGFβ‐induced CAGA‐dynGFP reporter activity in NIH3T3 control clones and betaglycan KO clones. Cells were pre‐treated with TGM6 (100 ng/ml) before stimulation with 1 ng/ml TGFβ for 21 h. Extended data for D and F are shown in Figure S3B,C, respectively.

In a search for TGM6 (co)receptors, we performed affinity crosslinking of iodinated TGM6 to cell surface proteins on responsive NIH3T3 cells (Figure 3A). Analysis of cell lysates immunoprecipitated with antibodies to TGFβ family type I (i.e., activin receptor‐like kinase (ALK)1, ‐2, ‐3, ‐4, ‐5 (TGFBR1), type 2 receptors (i.e., activin type II receptor (ACTR2)A, ACTR2B, TGFBR2, bone morphogenetic protein type II receptor (BMPR2) and type III receptors (i.e., endoglin and betaglycan)), revealed that as expected, only TGFBR2 from the type II receptors bound TGM6, but that none of the type I receptors or other type II receptors bound (Figure 3B). Interestingly, a betaglycan‐TGM6, but not endoglin‐TGM6, crosslinked complex was detected (Figure 3B). Importantly, in the TGM6 affinity‐labeled cell lysate, besides the TGFBR2‐TGM6 and betaglycan‐TGM6 complex, an additional prominent TGM6 protein complex of about 600 kDa was present (Figure 3B). These results suggest that betaglycan and another unknown 600 kDa protein are co‐receptors for TGM6 in NIH3T3 cells.

We found that TGM6 binds with high affinity (15 nM) to mTGFBR2, and in the crystal structure of the TGM6‐D3‐mTGFBR2 complex, the interface is remarkably similar to that of TGFβ‐TGFBR2 [19] (Figure 2). Moreover, affinity binding assays with purified proteins revealed that TGM6 D3 competes with TGFβ for binding to TGFBR2 [19]. We therefore investigated whether TGM6 can compete with TGFβ for mTGFBR on intact cells using affinity labeling of NIH3T3 cell surface proteins with iodinated TGFβ in the absence or presence of different amounts of TGM6. Cell lysates immunoprecipitated with TGFBR1, TGFBR2, or betaglycan‐specific antibodies were analyzed. Of note, these affinity labeling experiments were conducted with NIH3T3 cells kept on ice, thereby preventing TGFβ‐induced TGFBR internalization. We observed that increasing amounts of TGM6 inhibited the TGFβ‐mTGFBR1 and TGFβ‐mTGFBR2 interactions, but not the TGFβ‐betaglycan interaction (Figure 3C). TGM6 binds to TGFBR2, not TGFBR1; however, because TGFβ binding to TGFBR1 requires TGFBR2 (4), TGM6 also decreases TGFβ‐mTGFBR1 interaction. The competition between TGM6 and TGFβ for TGFBR binding is consistent with the function of TGM6 as a TGFβ antagonist.

### 2.4 Betaglycan Inhibits TGM6‐Induced Antagonism of TGFβ Signaling

To address the functional role of TGM6‐betaglycan interaction, we derived replicate knock‐out clones (1A, 1B, 2A, and 2B; 1 and 2 are corresponding to different guide RNAs, and A and B refer to different independent clones) for betaglycan in NIH3T3 cells using CRISPR‐CAS9 gene editing (Figure S3A). When we analyzed the TGM6‐induced repression of TGFβ‐induced SMAD2 phosphorylation (Figures 3D,E and S3B) and TGFβ‐induced SMAD3/4 transcriptional response and in control versus betaglycan knock‐out clones (Figures 3F and S3C), we observed that deficiency of betaglycan greatly potentiated the TGM6‐induced inhibitory effect on TGFβ signaling. Thus, betaglycan is a negative regulator of TGM6.

Betaglycan comprises two major domains: an N‐terminal Orphan domain and a C‐terminal Zona Pellucida (ZP) domain [25]. To map which betaglycan domain(s) interact(s) with TGM6, we used rat (r) L6E9 myoblast cells deficient in betaglycan. L6E9 myoblasts are responsive to TGM6 (Figure 1A). We first confirmed that mbetaglycan and rbetaglycan are expressed after transfection with expression plasmids (Figure S3D) and bind TGM6 in L6E9 cells (Figure S3E). Ectopic expression of rBetaglycan wildtype or mutants lacking either the Orphan or ZP domains (Figure S3F) all interfered with the ability of TGM6 to exert its inhibitory effect on TGFβ‐induced SMAD3/4 transcriptional response (Figure S3G) and TGFβ‐induced SMAD2 phosphorylation (Figure S3H). Thus, both orphan and ZP domains are important for TGM6‐Betaglycan interplay.

### 2.5 Identification of Low‐Density Lipoprotein Receptor‐Related Protein (LRP)1 as TGM6 Co‐Receptor, Which Is a Key Determinant for TGM6‐Induced Antagonism of TGFβ Signaling

**FIGURE 4:**
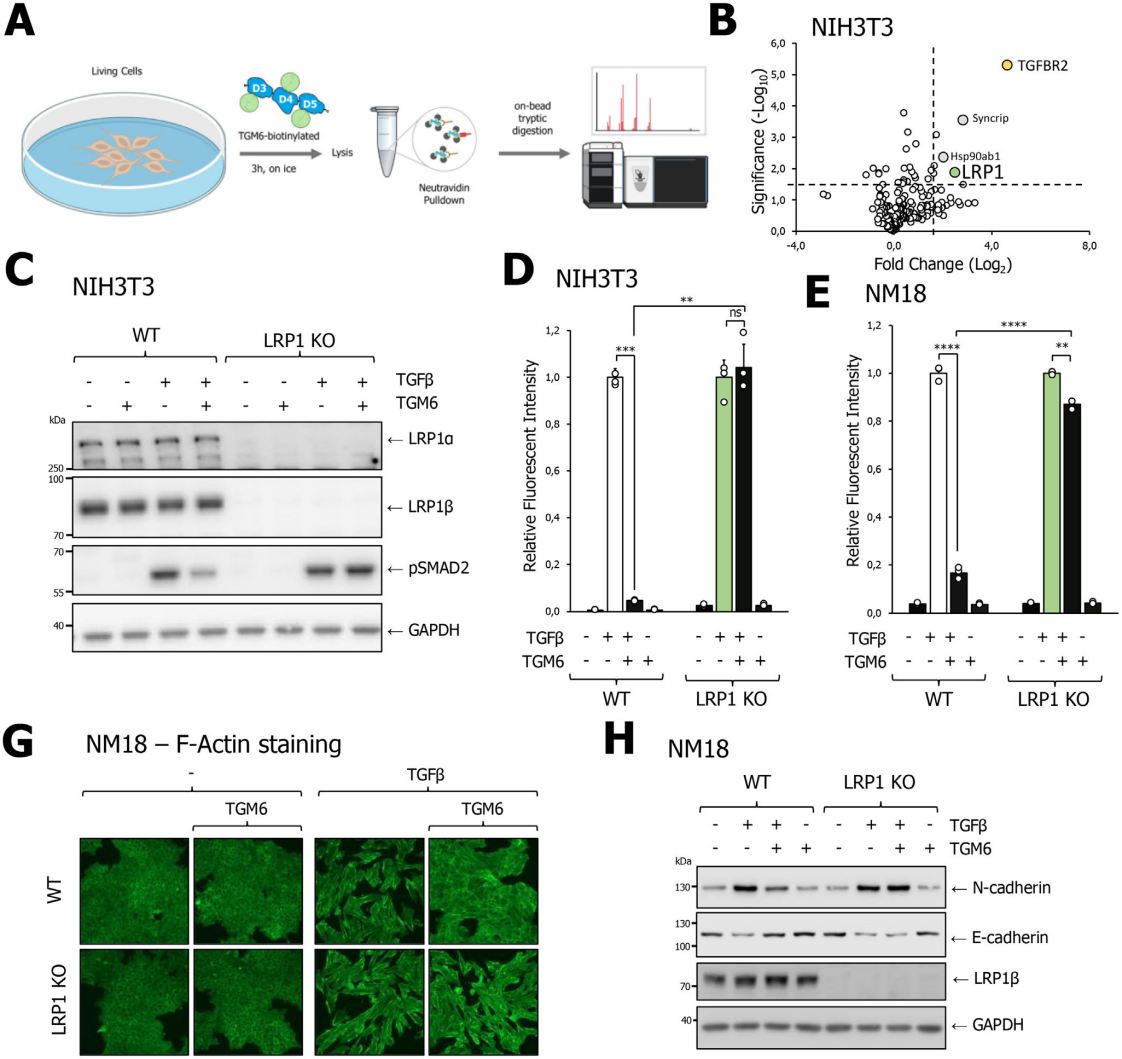
Identification of LRP1 as TGM6 co‐receptor. (A) Schematic representation of the experimental flow for the identification of TGM6 interactors using biotinylated TGM6. NIH3T3 cells were incubated with biotinylated TGM6, cells were lysed, and TGM6‐biotin and interaction partners were precipitated using neutravidin beads and analysed by mass spectrometry. (B) Analysis of TGM6 binding partners in NIH3T3 cells by mass spectrometry. Fold change is calculated relative to control cells undergoing the same procedure without TGM6‐biotin addition. TGM6 interaction partners TGFBR2 and LRP1 are highlighted with yellow and green circles. (C) Western blot analysis for the TGM6 effect on TGFβ‐induced SMAD2 phosphorylation in wild‐type NIH3T3 cells or derivatives deficient in LRP1. (D and E) Effect of LRP1 deficiency in NIH3T3 (D) and NM18 (E) cells on antagonism of TGM6 on TGFβ‐induced SMAD3 transcriptional activity. Extended data for 4C–E are shown in Figure S4C–F, respectively. (G and H) Effect of LRP1 deficiency on antagonism of TGM6 on TGFβ‐induced morphological transition and actin stress fiber formation (measured by phalloidin‐Alexa488 staining after 2‐days) (G) and epithelial E‐Cadherin /mesenchymal N‐Cadherin marker expression in NM18 cells (H). TGM6 and TGFβ were used at 100 and 1 ng/ml, respectively.

To identify cell‐surface binding proteins in an unbiased manner, biotinylated TGM6 was added to NIH3T3 cells, and the cell lysates were subjected to a pulldown assay using neutravidin beads. The bound proteins were trypsinized, and peptide fragments were analyzed by mass spectrometry (Figure 4A). As expected, TGFBR2, known to interact with TGM6, was identified as a highly abundant protein in the pulldown (Figure 4B). Among the other hits, low‐density lipoprotein receptor‐related protein 1 (LRP1) caught our attention (Figure 4B), as it is a large 600 kDa type I receptor glycoprotein involved in receptor‐mediated endocytosis [26]. Like other members of the LDLR protein family, LRP1 contains cysteine‐rich complement‐type repeats, epidermal growth factor (EGF) repeats, and β propeller domains, a transmembrane domain, and an intracellular domain. LRP1 contains four ligand‐binding domains numbered I to IV and multiple cysteine‐rich complement‐type repeats. LRP1 is processed by furin, thereby producing two non‐covalently bound chains. i.e., a 515 kDa α‐chain and an 85 kDa β‐chain [26]. Notably, the 600 kDa TGM6‐protein complex that was detected in the cell lysate (Figure 3B) corresponds to the expected size of TGM6 cross‐linked with (heavily glycosylated) LRP1α (and LRP1β).

Similar to our strategy for betaglycan, we investigated the functional role of the TGM6‐LRP1 interaction by knocking out LRP1 in NIH3T3 cells and NM18 cells using CRISPR‐CAS9 gene editing. We generated 4 LRP1 knockout clones (KO1A, KO1B, KO2A, and KO2B) in NIH3T3 cells (Figure S4A) and in knockout LRP1 pools (KO1 and KO2) of NM18 cells (Figure S4B). KO1A and KO1B were generated using different guides, and A and B refer to independent clones/pools. TGM6 was found to lose all inhibitory effects on the TGFβ‐induced pSMAD2 phosphorylation in LRP1‐KO cells (Figures 4C and S4C,D), a result confirmed by the intact TGFβ/SMAD3 transcriptional response in LRP1‐KO cells in the presence of TGM6 (Figure 4D, S4E). Similar results were obtained using NMuMG breast epithelial cells, clone NM18 (Figures 4E and S4F). NMuMG (NM18) cells are a frequently used model system for examining TGF‐β‐induced EMT [18]. The TGFβ‐induced morphological changes and formation of actin stress fibers observed in control cells were blocked by TGM6 in control/wild‐type cells, but not in LRP1‐deficient NM18 cells (Figure 4G). Consistently, whereas TGM6 blocked the downregulation of the epithelial marker E‐cadherin and the upregulation of the mesenchymal marker N‐cadherin in control/wild‐type cells, this was not observed in LRP1 knock‐out cells (Figure 4H). Thus, LRP1 is a critical determinant of the cell‐type‐specific potency of TGM6, presumably by increasing its cellular avidity relative to cell types that lack LRP1 expression.

### 2.6 TGM6 Interacts via Separate Domains With LRP1 and TGFBR2, and TGM6‐Betaglycan Requires TGFBR2

**FIGURE 5:**
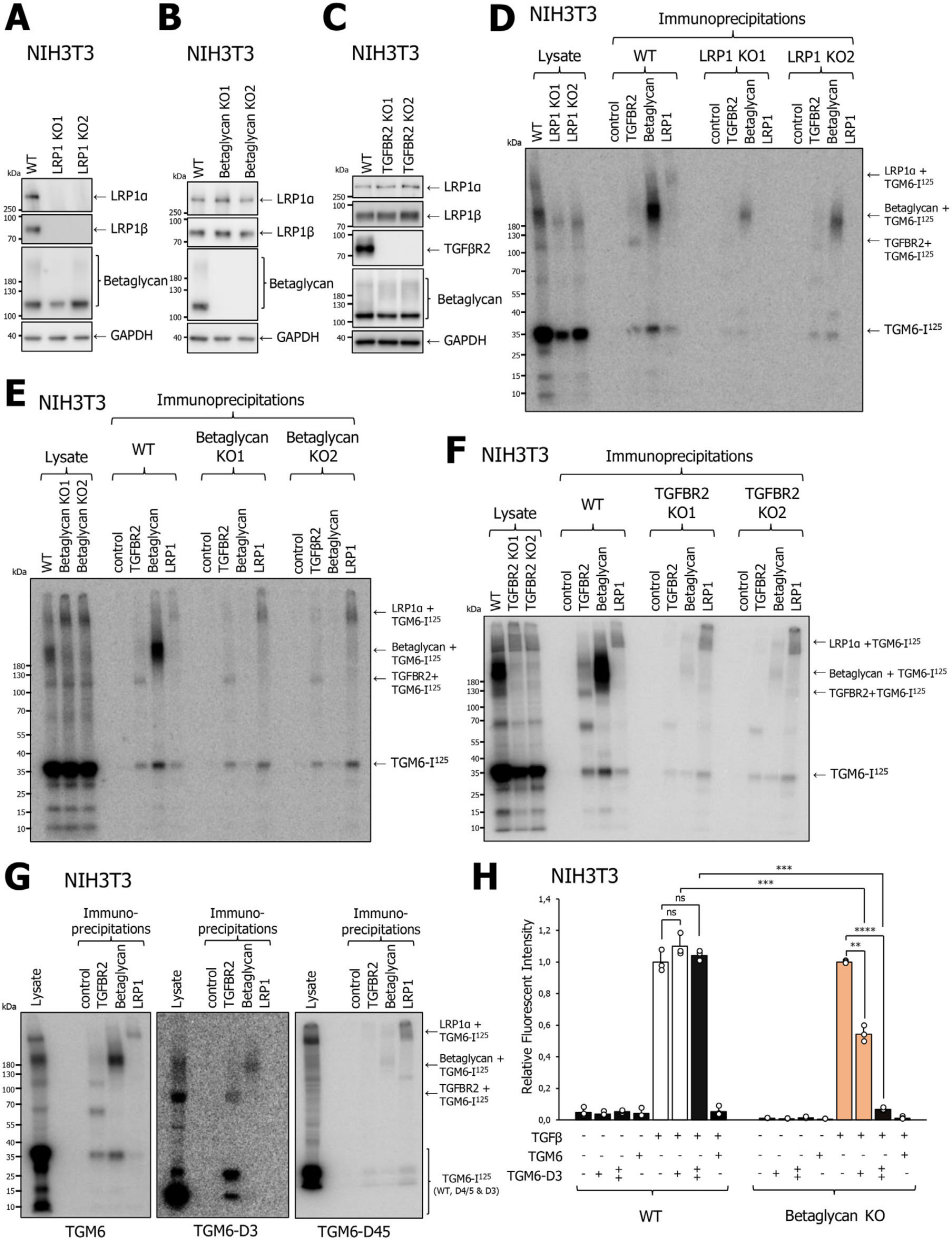
TGM6 D4/5‐LRP1 interaction occurs independently of TGBR2 and Betaglycan, but the interaction of TGM D3‐Betaglycan requires TGFBR2. (A‐C) Western blot analysis of LRP1, betaglycan, and TGFBR2 expression in NIH3T3 LRP1 (A), NIH3T3 betaglycan (B), or NIH3T3 TGFBR2 (C) knock‐out clones (1 and 2). (D‐F) Effect of LRP1 deficiency (D), betaglycan deficiency (E), or TGFBR2 deficiency (F) on TGM6 binding to (co)receptors. Iodinated TGM6 was cross‐linked to NIH3T3 control cells or various KO cells, and cell lysates were immunoprecipitated with the indicated antibodies. Total cell lysates were analysed. Signals were analysed by autoradiography. (G) Analysis of the binding of wild‐type TGM6, TGM6‐D3, and TGM6‐D4/5 to (co)receptors. Iodinated TGM6 (left panel), TGM6‐D3 (middle panel), or TGM6‐D4/5 (right panel) were used to affinity label cell surface proteins on NIH3T3 cells, and cell lysates were immunoprecipitated using the indicated antibodies. Total cell lysates were also analysed. Signals were analysed by autoradiography. (H). Effect of betaglycan deficiency on the ability of TGM6‐D3 to antagonize TGFβ‐induced CAGA‐dynGFP reporter activity in NIH3T3 and betaglycan NIH3T3 KO cells. Cells were pre‐treated with TGM6 (100 ng/ml) or TGM6‐D3 (1000 or 5000 ng/ml) for 30 min before stimulation with 1 ng/ml TGFβ for 21 h. Extended data for **5H** is shown in Figure S5.

To further validate the TGM6 interaction with LRP1 and investigate the possible interplay by which the three different receptors and co‐receptors interact with TGM6, we compared the binding of TGM6 to cell surface proteins in wild‐type NIH3T3 cells or NIH3T3 cells deficient in LRP1, betaglycan, or TGFBR2 (Figure 5A–C). Affinity labeling with iodinated TGM6 in control/wild‐type cells, followed by immunoprecipitation of cell lysates with an LRP1 antibody, confirmed that TGM6 binds LRP1. Immunoprecipitation for TGFBR2 or betaglycan in cell lysates of LRP1 knock‐out cells that were affinity labeled with iodinated TGM6 revealed that TGM6, in the absence of LRP1, is still able to interact with TGFBR2 and betaglycan, albeit weaker than in control/wild‐type cells (Figure 5D). We observed no co‐immunoprecipitation of TGFBR2 or betaglycan with LRP1, indicating no heteromeric complex formation (Figure 5D). Similar affinity labeling followed by receptor immunoprecipitation experiments performed with iodinated TGM6 on NIH3T3 betaglycan knock‐out cells or control/wild‐type cells showed that TGM6 binding to TGFBR2 is not different in knock‐out versus control/wild‐type cells, while TGM6 binding to LRP1 is slightly increased in betaglycan‐deficient cells (Figure 5E). Affinity labeling of cell‐surface receptors with iodinated TGM6 on NIH3T3 cells deficient in TGFBR2, versus control/wild‐type, revealed that betaglycan binding is nearly absent and that the TGM6‐LRP1 interaction is unperturbed (Figure 5F). Thus, TGM6 binds TGFBR2 and LRP1 without needing betaglycan, but betaglycan requires TGFBR2 to interact with TGM6.

To further consolidate and expand these findings, we compared the binding of intact TGM6, TGM6‐D3, or TGM6‐D4/5 truncations to the three (co)receptors (Figure 5G). As expected, intact TGM6 interacts with TGFBR2, betaglycan, and LRP1. TGM6‐D3 weakly interacts with TGFBR2 and betaglycan (and not LRP1), and TGM6‐D4/5 interacts with LRP1 (and not TGFBR2 and betaglycan) (Figure 5G). Thus, TGM6‐D3 is both required and sufficient for interaction with TGFBR2 and betaglycan, while TGM6‐D4/5 is essential and sufficient for LRP1 binding.

We previously demonstrated that TGM6‐D3 is insufficient to inhibit TGF‐β signaling in fibroblasts [19]. As betaglycan inhibits TGM6 function, we compared the effect of TGM6‐D3 in wild‐type versus betaglycan knock‐out cells to investigate if, under these conditions, TGM6‐D3 can exert TGFβ antagonism. TGM6‐D3 inhibited the TGFβ‐induced SMAD3 transcriptional response in a dose‐dependent manner, albeit with very high doses of TGM6 (1000 to 5000 ng/ml) in NIH3T3 cells depleted of betaglycan, but not in wild‐type NIH3T3 cells (Figures 5H and S5).

### 2.7 TGM6 D4/5 Interacts With LRP1 via its LDLaIV Cluster

**FIGURE 6:**
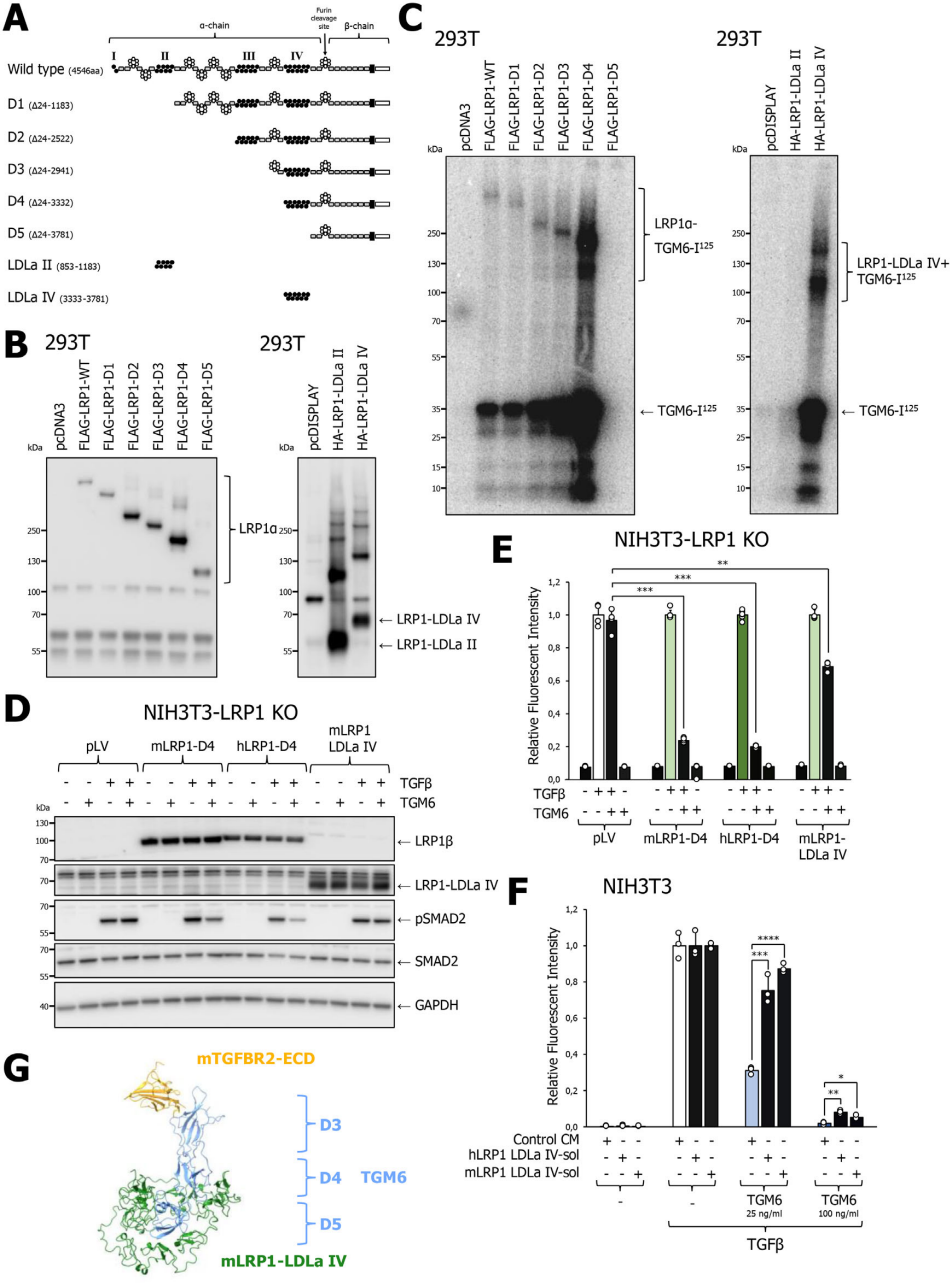
LRP1 LDLaIV cluster interacts with TGM6‐D4/5. (A) Schematic of the LRP1 constructs, WT, D1, D2, D3, D4, and D5 (pcDNA3), LDLaII, and LDLaIV (pDISPLAY). This figure was modified from [58]. (B) Expression analysis of LRP1 wild‐type and deletion constructs by Western blot analysis of proteins that were analysed by immunoprecipitation in (C). (C) Analysis of TGM6 binding to LRP1 deletion constructs. HEK293T cells overexpressing the different constructs were used to cross‐link with radio‐labeled TGM6. LRP1 was immunoprecipitated using anti‐FLAG (deletion constructs on the left) or anti‐HA (LDLa clusters on the right). (D and E) Effect of hLRP1‐D4, mLRP1‐D4, and mLRP1 LDLaIV on TGM6‐induced inhibition of TGFβ signaling in LRP1 KO cells as measured by SMAD2 phosphorylation (D) and CAGA‐dynGFP reporter activation (E). Extended quantification results for 5D are shown in Figure S6A. (F) Effect of soluble m LRP1‐LDLaIV and hLRP1‐LDLaIV on TGM6‐induced inhibition of TGFβ‐induced CAGA‐dynGFP reporter activation. TGM6 was pre‐incubated with conditioned media containing soluble LRP1 LDLaIV for 30 min. Subsequently, this was used to pre‐incubate NIH3T3 cells for 30 min, followed by stimulation with TGFβ for 21 h. (G) AlphaFold prediction of TGM6 in complex with mTGFBR2 ECD and mLRP1‐LDLaIV, showing independent binding of TGM6‐D3 to mTGFBR2 and TGM6‐D4/5 to mLRP1‐LDLaIV. TGM6 and TGFβ were used at 100 and 1 ng/ml, respectively.

To map the LRP1 domain that interacts with TGM6 D4/5, we generated a series of mLRP1 deletion constructs. All mLRP1 deletion constructs retained a signal peptide and a transmembrane domain to enable cell surface expression (Figure 6A). Expression of LRP1 constructs after transfection of 293T cells was confirmed by Western Blot analysis of cell lysates (Figure 6B). Affinity labeling of LRP1 (deletion) expression constructs with iodinated TGM6 revealed that TGM6 binds to LRP1‐D4 (Figure 6C, image on the left). This region contains the LDLaIV cluster, of which the tandem repeats of the cysteine‐rich lipoprotein receptor class A (LDLa) can form binding sites for ligands [26]. TGM6 was found to interact with the LDLaIV cluster, but not with a related LDLaII cluster used as a specificity control (Figure 6C, image on the right). Ectopic expression of hLRP1 or mLRP1 D4 in LRP1‐deficient NIH3T3 cells rescued the ability of TGM6 to inhibit TGFβ signaling as measured by TGFβ‐induced SMAD2 phosphorylation (Figures 6D and S6A) and SMAD3‐mediated transcriptional response (Figure 6E). Furthermore, we found that mLRP1‐LDLaIV (with the transmembrane (TM) domain) rescued the TGM6‐inhibitory effect on TGFβ/SMAD signaling, albeit less efficiently than mLRP1‐D4 (Figure 6D,E). Next, we investigated the impact of soluble mouse or human LRP1‐LDLaIV on TGM6‐induced inhibition of TGFβ/SMAD signaling. Both soluble proteins were equally expressed in the conditioned media of transfected HEK293T cells (Figure S6B). We found that soluble hLRP1‐LDLaIV or mLRP1‐LDLaIV inhibits the TGM6 inhibitory effect on TGFβ/SMAD3 transcriptional response in a dose‐dependent manner (Figures 6F and S6C). LRP1‐LDLaIV acts as a trap, sequestering TGM6, preventing its interaction with LRP1. Our results support the notion that the differential binding of TGM6 to mTGFBR2 versus hTGFBR2 (Figure 2), rather than to mLRP1 versus hLRP1, is the critical determinant of mouse versus human cell selectivity of TGM6 responses. Consistent with our biochemical results, Alphafold3 predicted with high confidence specific interactions between TGM6‐D3 and TGM6 D4/5 with mTGFBR2 and mLRP1‐LDLa IV, respectively (Figure 6G). Taken together, our results indicate that the LDLaIV cluster is both sufficient and required for TGM6 interaction, and that TGM6‐D4/5 interaction with LRP1 occurs independently of TGM6‐D3.

### 2.8 TGM6 Antagonizes TGFβ Signaling by Inducing LRP1‐Dependent TGFBR2 Lysosomal Degradation

**FIGURE 7:**
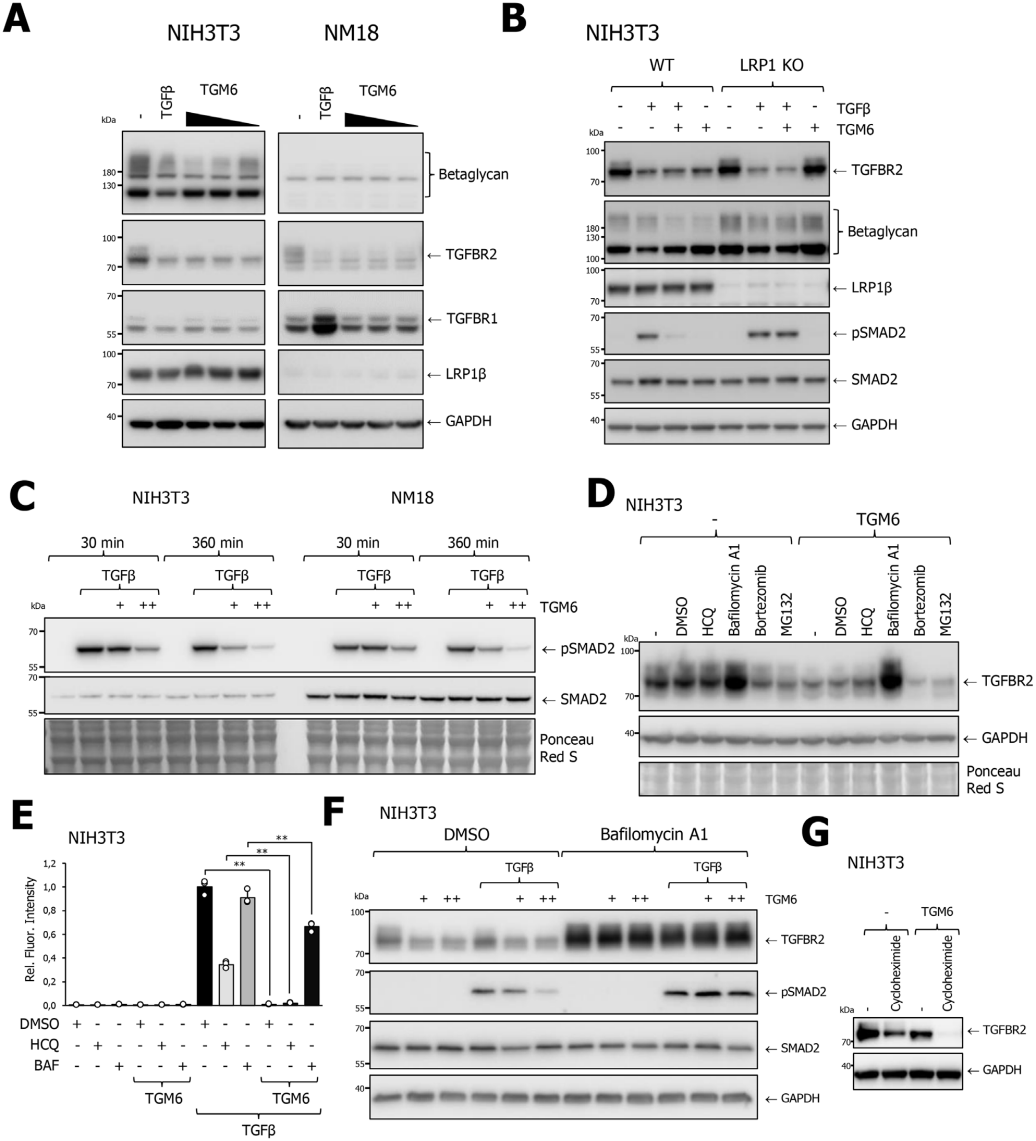
TGM6 antagonizes TGFβ signaling by inducing LRP1‐dependent TGFBR2 lysosomal degradation. (A) Effect of TGM6 on expression of betaglycan, TGFBR1, TGFBR2 and LRP1β in NIH3T3 (Left) and NM18 cells (Right). Cells were treated with TGM6 (5, 50, or 500 ng/ml) or TGFβ for 6 h. The two panels in (A) were run on the same gel and analysed in the same manner. (B) Effect of TGM6 or TGFβ on expression of TGFBR2, betaglycan, TGFBR1, LRP1β, phosphorylated SMAD2 (pSMAD2), and SMAD2 expression levels in control /wild‐type and LRP1 KO NIH3T3 cells. Cells were pre‐treated with TGM6 for 30 min (where indicated) and stimulated with TGFβ (where indicated) for 6 h. (C) Effect of TGM6 on TGFβ‐induced SMAD2 phosphorylation in NIH3T3 cells. TGM6 was added either 30 min or 360 min prior to the addition of TGFβ. Quantification of results in C is shown in Figure S7A. (D) Effect of DMSO vehicle control, hydroxychloroquine (HCQ, 20 µM), bafilomycin A1 (20 nM), bortezomib (10 nM), and MG132 (5 µM) on TGM6‐induced downregulation of TGFBR2 expression. (E) Effect of TGM6 on TGFβ‐induced SMAD3 transcriptional response in the presence of DMSO vehicle control, hydroxychloroquine (HCQ), or bafilomycin A1. The CAGA‐dynGFP transcriptional response was measured after 20 h. (F) Effect of inhibition of TGM6 on TGFβ‐induced SMAD2 phosphorylation in the absence or presence of bafilomycin A1. Pretreatment with TGM6 lasted 3 h, and subsequent TGFβ treatment lasted 3 h. Extended results for F are shown in Figure S7B. (G) Effect of TGM6 and/or cycloheximide (25 µg/ml) on TGFR2 expression, cells were pretreated with cycloheximide for 30 min before TGM6 (2 h). In A‐D, F, G, the protein expression was analysed by Western blotting. In D‐F, compounds or vehicle control were added 1 h before ligand stimulation. TGM6 and TGFβ were used at 100 ng/ml and 1 ng/ml, respectively (unless indicated otherwise).

LRP1 is involved in receptor endocytosis and in lysosomal degradation [26]. We therefore set out to explore whether TGM6 engagement with TGFBR2 and LRP1 induces TGFBR2 degradation. Challenging NIH3T3 and NM18 cells with TGM6 decreased TGFBR2, but not TGFBR1 levels (Figure 7A). No effect of TGM6 on LRP1 expression was found. TGM6 stimulation decreased mature cell‐surface‐associated betaglycan (with a molecular weight of 140‐250 kDa), but did not affect immature intracellular betaglycan (with a molecular weight of 100 kDa) levels in NIH3T3 cells. No effect on betaglycan expression was found in NM18 cells (Figure 7A). The agonist TGFβ inhibited TGFBR2 in both cell types; thus, TGFBR2 downregulation is not per se associated with an antagonistic effect. TGFβ stimulated TGFBR1 levels in NM18 cells (Figure 7A). Next, we examined the impact of TGM6 and/or TGFβ on TGFBR2 levels in control and LRP1‐deficient NIH3T3 cells. We observed that TGM6 and TGFβ inhibit TGFBR2 expression in control/wild‐type cells (Figure 7B). In LRP1‐deficient cells, TGM6 was unable to downregulate TGFBR2, but TGFβ remained proficient in TGFBR2 downregulation (Figure 7B). Consistent with these results, we observed that TGM6 inhibits SMAD2 phosphorylation, and that this effect is more pronounced if the TGFβ agonist is added after 360 min rather than 30 min in NH3T3 and NM18 cells (Figures 7C and S7A). While competition between TGFβ and TGM6 for TGFBR2 occurs immediately, the TGM6‐induced TGFBR2 downregulation is more pronounced at 6 h than at 1 h of treatment.

Next, we examined the effects of bafilomycin A1 (a lysosomal inhibitor that directly inhibits the pump that transfers protons into the lysosome, leading to increased lysosomal pH), hydroxychloroquine (is an autophagy inhibitor that acts at the late stage by blocking the fusion of autophagosomes with lysosomes), bortezomib and MG132 (proteasome inhibitors) on TGM6‐induced reduction in TGFBR2 levels in NIH3T3 cells. We found that treatment with bafilomycin A1, but not proteasome inhibitors, potently blocked the TGM6‐induced decrease in TGFBR2 expression. Hydroxychloroquine only weakly inhibited the decrease in TGM6‐induced TGFBR2 expression; this is consistent with the notion that bafilomycin A1 is a direct lysosomal inhibitor, whereas hydroxychloroquine enters the lysosome and neutralizes the acid (Figure 7D). In the presence of bafilomycin A1 and in the absence of TGM6, basal TGFBR2 levels were increased (Figure 7D), compatible with a previous report that TGFBR2 stability is controlled by lysosomal, but not proteasomal, function [27]. Moreover, bafilomycin nearly completely counteracted the inhibition of TGM6 on TGFβ‐induced SMAD3 reporter activity (Figure 7E) and SMAD2 phosphorylation (Figures 7F and S7B) in NIH3T3 cells. Furthermore, co‐treatment of NIH3T3 cells with TGM6 and cycloheximide, an inhibitor of protein synthesis, reduced TGFBR2 levels more than either agent alone (Figure 7G). Taken together, our results reveal that in addition to competition between TGM6 and TGFβ for TGFBR2 (Figure 3C), TGM6 antagonizes TGFBR2 signaling by mediating LRP1‐dependent TGFBR2 lysosomal degradation (Figure 7D–G).

### 2.9 TGM6 Acts in *Cis* and Not in *Trans*

TGM6 has the D3 and D4/5 domains that interact simultaneously but independently with TGFBR2 and LRP1, respectively. This allows them to engage target TGFBR2^+^LRP1^+^ cell types in*‐cis* with high cellular selectivity and avidity. The modular design may also provide sufficient flexibility for TGM6 to interact with TGFBR2 and LRP1 across different cell types and act in*‐trans*, thereby inhibiting TGFβ signaling in LRP^−^ cells that neighbor LRP1^+^ cells. To study if TGM6 acts in*‐cis* and/or in*‐trans*, we used NIH3T3‐CAGA‐dynGFP cells (TGFBR2^+^LRP1^+^) and NIH3T3‐CAGA‐mCHERRYd2 deficient in LRP1 (TGFBR2^+^LRP1^−^), either as mono‐ or mixed cultures in two different ratios (Figure S8A), and challenged them with TGM6 and/or TGFβ. As NIH3T3‐CAGA‐dynGFP and NIH3T3‐CAGA‐mCHERRYd2 express TGFBRs, TGFβ will induce CAGA‐driven reporter activity in the two NIH3T3 cell types equally. Analysis of CAGA‐dynGFP and CAGA‐mCHERRYd2 levels revealed that, as expected, TGM6 potently inhibited TGFβ‐induced CAGA‐dynGFP response in the absence or presence of TGFBR2^+^LRP1^−^ cells, and TGM6 did not inhibit TGFβ‐induced CAGA‐mCHERRYd2 response in monoculture of TGFBR2^+^LRP1^−^ cells. CAGA‐mCHERRYd2 signals were not inhibited when NIH3T3‐CAGA‐dynGFP and NIH3T3‐CAGA‐mCHERRYd2 were co‐cultured (Figure S8B,C). To increase interactions between NIH3T3‐CAGA‐mCHERRYd2 and NIH3T3‐CAGA‐dynGFP, we used a five‐fold excess of NIH3T3‐CAGA‐mCHERRYd2 over NIH3T3‐CAGA‐dynGFP. As CAGA‐dynGFP is still potently decreased by TGM6 under these latter conditions, TGFBR2 in LRP1‐deficient cells does not act as a sink to prevent TGM6 engagement with TGFBR2 on LRP1^+^ cells. These results suggest that TGM6 only acts in*‐cis* and is effective on LRP1‐expressing target cells, but not on co‐cultured LRP1^−^TGFBR2^+^ cells (Figure S8B,C).

### 2.10 Engineering TGM6‐TGM1 Chimeras to Switch Cell‐Type‐Specificity and Change Functionality

**FIGURE 8:**
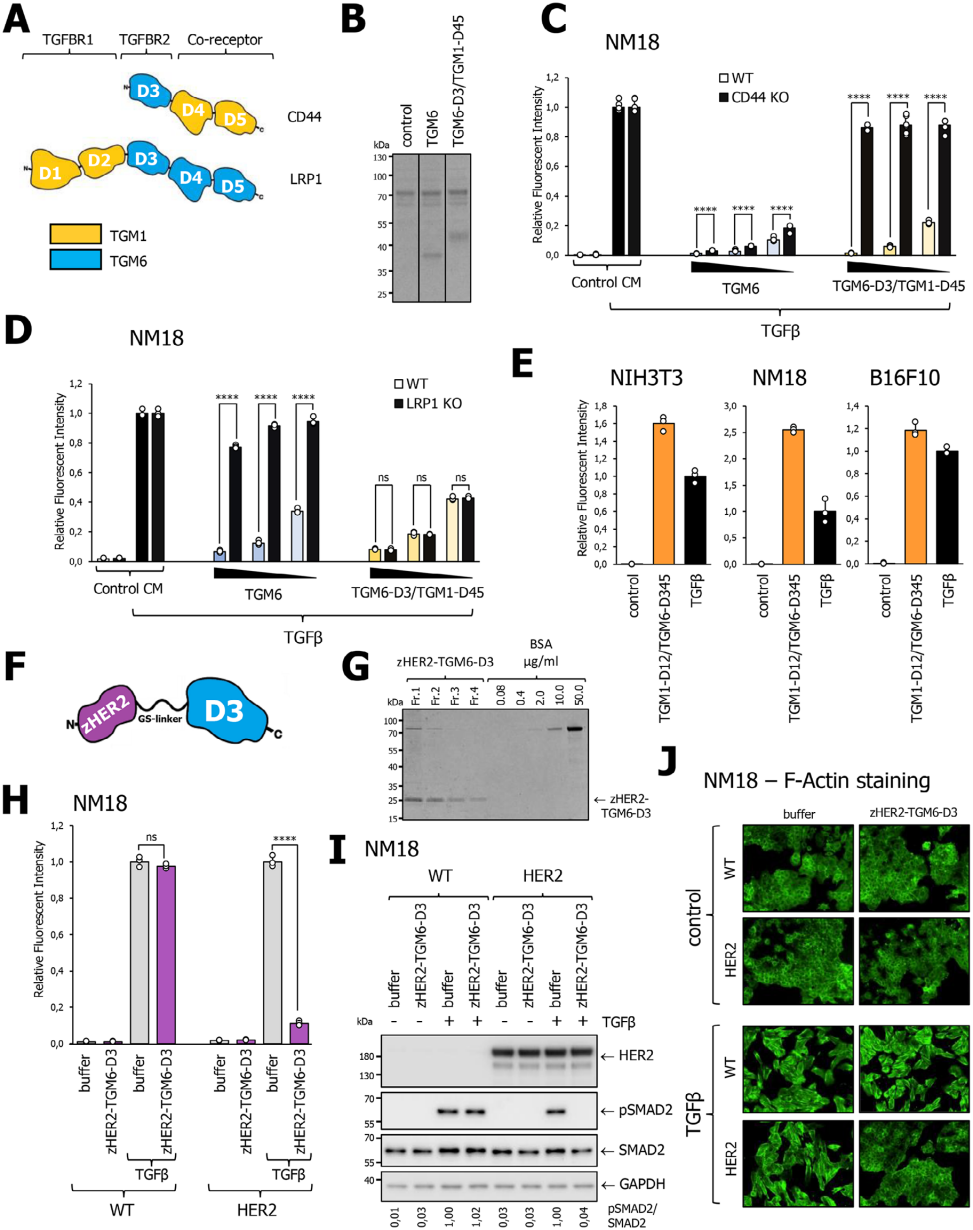
TGM6 chimeras change cell specificity and change TGM6 antagonist into a potent TGFβ agonist. (A) Schematic of the TGM1‐TGM6 fusion proteins that were generated. (B) Coomassie stain of SDS polyacrylamide gel to analyze the expression and purity of TGM proteins and fusions in the conditioned media of transfected 293T cells. All three samples were run on the same gel. Lines indicate where the gel was cut. (C, D) Effect of TGM6 or TGM6‐D3‐TGM1‐D45 chimera on the inhibitory potential of TGFβ signaling in (C) NM18 CAGA‐mCHERRYd2 WT vs CD44 KO cells (single cell clone) and (D) NM18 CAGA‐mCHERRYd2 WT vs LRP1 KO cells (pool). (E) Effect of TGM1‐D12‐TGM6 chimera or TGFβ in NIH3T3, NM18, and B16F10 CAGA‐dynGFP cells. (F). Schematic of the zHER2‐TGM6 fusion protein. (G) Coomassie stain of SDS polyacrylamide gel to analyze the expression and purity of the produced zHER2‐TGM6 fusion protein (Consecutive fractions 1 to 4 were pooled). Different amounts of BSA were used to estimate protein concentration. (H, I and J) Effect of zHER2‐TGM6 fusion protein on SMAD3‐induced transcriptional reporter activity (H), TGFβ‐induced SMAD2 phosphorylation (as analysed by Western blotting) (I), and stress fiber formation (as analysed by F‐actin staining) (J) in NM‐18 wild‐type or HER2 overexpression cells. The pSMAD2/SMAD2 ratios are indicated underneath GAPDH blot results in I. Stress fibers were visualized by phalloidin‐Alexa488 staining. zHER2‐TGM6 (40 nM) was added 30 min before the addition of TGFβ. As a vehicle control elution buffer was used. For SMAD2 phosphorylation, transcriptional, and stress fiber formation assays, cells were treated for 1 h, 15 h, or 2 days, respectively. TGFβ was used at 1 ng/ml.

The modular structure of TGMs prompted us to generate TGM chimeras to determine whether we could alter cell selectivity and function. TGM1 mimics TGFβ's effects on CD44‐expressing cells, and TGM6 antagonizes TGFβ signaling in LRP1‐expressing cells. We generated a chimera in which we switched TGM6‐D4/5 for TGM1‐D4/5 (termed TGM6‐D3/TGM1‐D4/5) (Figure 8A). Conditioned media of 293T cells transfected with an expression plasmid (or empty vector for control) were used to stimulate cells (Figure 8B). When TGM6 and TGM6‐D3/TGM1‐D4/5 were tested in NM18 or NM18 cells deficient in CD44, we found that TGM6‐D3/TGM1‐D4/5 mimicked TGM6 in inhibiting TGFβ signaling in control/wild‐type NM18 cells but not in CD44‐deficient NM18 cells (Figure 8C). Whereas LRP1 is essential for TGM6 to inhibit TGFβ signaling in NM18 cells, TGM6‐D3/TGM1‐D4/5 inhibited equally TGFβ responses in wild‐type/control or LRP1‐deficient cells (Figure 8D). We thus changed the TGFβ‐antagonistic cell specificity of wild‐type TGM6 from LRP^+^ cells to CD44^+^ cells by using the chimera TGM6‐D3/TGM1‐D4/5 to inhibit TGFβ signaling in CD44^+^ cells.

In another chimera, we fused TGM1 1D1/2 onto the N‐terminus of TGM6 (termed TGM1‐D1/2‐TGM6) (Figure 8A). When this TGM chimera protein or TGFβ was used to challenge NM18 cells, we found that the TGM1‐D1/2‐TGM6 chimera and TGFβ potently activated the TGFβ/SMAD3 transcriptional response. We thus altered the TGM6 antagonist into a TGM1‐D1/2‐TGM6 agonist (Figure 8E).

Next, to explore the possibility of expanding the targeting of TGMs to fusions with an affibody/nanobody recognizing other cell surface receptors, we fused TGM6‐D3 via a short linker sequence to an affibody [28] that engages with the human epidermal growth factor receptor 2 (HER2), and termed this zHER2‐TGM6‐D3 (Figure 8F). We expressed and purified the zHER2‐TGM6‐D3 protein (Figure 8G). Whereas zHER2‐TGM6‐D3 does not affect TGFβ‐induced SMAD3 transcriptional response and SMAD2 phosphorylation in NM18 cells, these responses are blocked by zHER2‐linkTGM6‐D3 in NM18‐HER2 overexpressing cells (Figure 8H,I). Furthermore, the TGFβ‐induced morphological change of NM18 cells and stress fiber formation were not affected by zHER2‐TGM6‐D3 in wild‐type cells but blocked in NM18‐HER2 expressing cells (Figure 8J). Thus, the modular structure of TGMs enabled us to engineer synthetic TGM1/6 chimeras and fusions, thereby modulating cell‐type specificity and functionality.

### 2.11 Engineering TGFBR2‐VHH‐Based Fusions for Cell‐Type‐Specific Targeting of TGFβ Signaling

**FIGURE 9:**
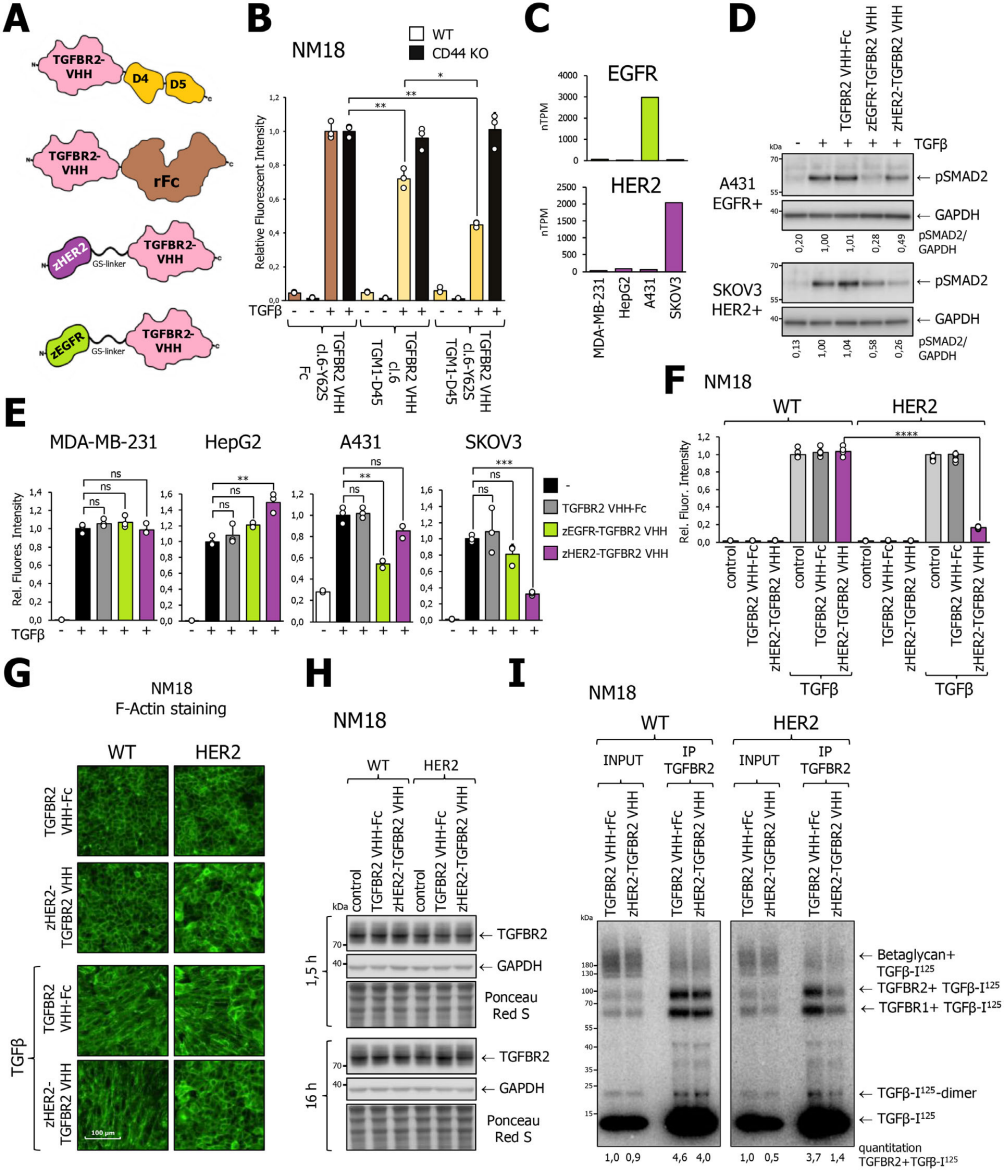
Designer TGFBR2 VHH‐based bispecific antibodies that inhibit TGF‐β receptor signaling in a cell‐type‐specific manner. (A) Schematic presentations of TGFBR2‐VHH‐based protein fusions that were generated.; zHER2 and zEGFR are HER2 and EGFR high‐affinity affibodies, respectively. (B) Effect of TGFBR VHH cl.6‐Y62S fused to TGM1‐D4/5 and control TGFBR2‐VHH‐cl.6‐Y62S fused to rFC on TGFβ/SMAD3 induced CAGA‐GFP transcriptional reporter activity in wild‐type or CD44 knock‐out NM18 cells stimulated with TGFβ. (C) Analysis of mRNA expression levels in nTPM (transcripts per million) of EGFR and HER2 in the indicated cancer cell lines using data from the Human Protein Atlas. (D and E) Effect of TGFBR2‐Fc, zEGFR‐TGFBR2 VHH, or zHER2‐TGFBR2 VHH on TGFβ‐induced SMAD2 phosphorylation (D) and on TGFβ‐induced SMAD3 transcriptional response (E) in the indicated cell lines. The pSMAD2/GAPDH ratios are indicated underneath GAPDH blot results in D. (F and G) Effect of zHER2‐TGFBR2 VHH or TGFBR2 VHH‐Fc on TGFβ−induced SMAD3 transcriptional response (F) and EMT (G) in NM‐18 wild‐type (WT) and HER2 overexpressing cells. F‐actin stress fibers were visualized by phalloidin‐Alexa488 staining. (H) Effect of zHER2‐TGFBR2 VHH or TGFBR2‐VHH‐Fc (1.5‐h or 16‐h treatment) on TGFBR2 expression in NM18 wild‐type and HER‐expressing NM18 cells, as analysed by Western blotting. (I) Effect of zHER2‐TGFBR2 VHH or TGFBR2‐VHH‐Fc on binding of iodinated TGFβ to cell surface proteins without and with TGFBR2 immunoprecipitation. The relative intensity of TGFBR2‐TGFβ‐I^125^ is indicated below the autoradiogram. In B‐G, TGFβ was used at 1 ng/ml. In panel (D‐F), TGFBR2‐VHH‐cl.6‐Y62S is used, and indicated as TGFBR2‐VHH.

As the affinity of TGM6 D3 is much higher for mouse than human TGFBR2 ECD, the TGM6‐D3‐based fusion constructs are highly effective in inhibiting TGFβ signaling in mouse cells, but likely less so in human cells. Moreover, despite the low immunogenicity of many secreted parasite proteins, repeated exposure in mammals can eventually elicit an immune response. This may limit the potential of parasite‐derived proteins as therapeutics due to their limited efficacy and safety. We therefore set out to design bispecific antibodies (BsAbs) without parasitic protein sequences that inhibit TGFβ signaling in a human cell‐type‐specific manner, similar to TGMs. We screened a phage display library for specific nanobody/variable heavy domain (VHH) binders to TGFBR2‐overexpressed in HEK 293 T cells, and performed subsequent affinity maturation by yeast display using recombinant TGFBR2 ECD (Figure S9A). Specific TGFBR2‐VHH binders were characterized by the efficiency of immunoprecipitation of TGFBR2 and FACS analysis. Clone 6 was identified as the strongest binder by immunoprecipitation (Figure S9B), and it was used in an affinity maturation assay to identify clone 6A as a high‐affinity binder (Figure S9C). A binding assay revealed that the apparent 32.5 nM affinity for TGFBR2 to clone 6 was increased to 87 pM for clone 6A (Figure S9D). The effect of ectopic TGFBR2 overexpression (without exogenous added ligand on CAGA‐GFP reporter activity) was slightly inhibited by clones 6A and 6‐Y62S, but not by clone 6 (Figure 9E). Clone 6A had three mutations as compared to clone 6, one of which (Y62S) was solely responsible for the improved affinity, and this single mutant was used in all further experiments. Comparing the crystal structures of TGFBR2 with hTGFβ3 (PDB 1KTZ) and AlphaFold predicted hTGFBR2 ECD with TGFBR2‐VHH revealed that the contact sites between the two protein pairs in the complexes overlap (Figure S9F). However, TGFBR2‐VHH, when fused to Fc, was unable to inhibit TGFβ signaling (Figure S9G). For the purity assessment of TGFBR2‐VHH‐based proteins, see Figure S10A‐C. The biochemical and functional properties of TGFBR2 VHH are reminiscent of TGM6‐D3, which, by itself, has high affinity for mTGFBR2 but cannot inhibit TGFβ signaling. We next fused TGFBR2‐VHH to TGM1‐D4/5 (Figure 9A) to determine whether this fusion could restore TGFβ‐inhibitory activity in a cell‐type‐specific manner. When tested for its effect on the TGFβ‐induced SMAD transcriptional response in wild‐type or CD44‐knockout NM18 cells, we found that TGFBR2‐VHH‐TGM1‐D4/5 phenocopied the differential antagonistic effect of TGM6‐D3‐TGM1‐D4/5 in wild‐type versus CD44‐depleted NM18 cells (Figure 9B). Next, we fused TGFBR2‐VHH to affibodies against HER2 (zHER2) [28] or EGFR (zEGFR) [29] (Figure 9A). These BsAbs were then tested on TGFβ‐induced SMAD2 phosphorylation and SMAD3 transcriptional response in various TGFβ‐responsive cell lines. Notably, EGFR and HER2 are selectively highly expressed in A431 lung adenocarcinoma and SKOV3 ovarian cancer cell lines, respectively (Figure 9C). Consistent with our expectation, zEGFR‐TGFBR2‐VHH and zHER2‐TGFBR2‐VHH inhibited TGFβ induced SMAD2 phosphorylation (Figure 9D) and SMAD3‐induced transcriptional activity (Figure 9E) selectively in A431 and SKOV3 cell lines, respectively. In addition, similarly to what we found for zHER2‐TGM6‐D3, zHER2‐TGFBR2 ‐VHH blocked TGFβ/SMAD‐induced SMAD3‐induced transcriptional response (Figure 9F) and EMT in NM18‐HER2 overexpressing, but not wild type NM18 cells (Figure 9G). Next, we wanted to explore the mechanism of action of zHER2‐TGFBR2 VHH. We found that zHER2‐TGFBR2 VHH does not reduce TGFBR2 expression, regardless of cellular HER2 status or stimulation time (1.5 or 16 h) (Figures 9H and S11). However, the zHER2‐TGFBR2 VHH (unlike TGFBR2‐VHH‐Fc) effectively inhibits TGFβ from binding to TGFBR2 in HER2‐expressing NM18 cells relative to wild‐type cells (Figure 9I). This explains how zHER2‐TGFBR2 acts as a TGFβ cell‐type‐specific antagonist through a HER2‐dependent mechanism.

## 3 Discussion

The murine helminth parasite‐secreted TGM cytokines form a family of 10 structurally related proteins that mimic or antagonize TGFβ‐induced responses in selected host cell types [18, 19, 20]. Here, we report the identification of TGM6 co‐receptors. TGM6 directly partners with its D4/5 to co‐receptor LRP1, which is essential for TGM6's antagonism. In addition, TGM6, via its D3, interacts with co‐receptor betaglycan, which attenuates the TGM6‐mediated inhibitory effect on TGFβ/SMAD signaling. The modular structure of TGMs enabled us to generate TGM chimeras and inspired us to develop TGFBR2‐VHH‐based BsAbs that modulate TGFβ signaling only in target co‐receptor‐expressing cells. We thus developed a toolbox of cell‐type‐restricted pharmacological manipulators of TGFβ/SMAD signaling.

A survey of mouse and human cell types from distinct tissues demonstrated that TGM6 could inhibit TGFβ signaling in mouse cells but not in human cells. As parasite TGMs have evolved through convergent evolution in mice, and not in humans [21], this provides a rationale for their mouse specificity. TGM6 interacts with TGFBR2 and LRP1 to antagonize TGFβ signaling. Our results indicate that TGM6 binding to LRP1 does not contribute to species‐specific differences in TGM6 activity. We observed that (1) the ectopic expression of mLRP1 or hLRP1 was equally efficient in rescuing the TGM6 inhibitory effect in NIH3T3 LRP1 knock‐out cells, and (2) that the mouse or human soluble LDLaIV cluster, i.e., the LRP1 interacting domain for TGM6, was equally effective in blocking the TGM6‐induced inhibition of TGFβ signaling. Our results revealed that TGFBR2 is the critical determinant for TGM6 species specificity. Mouse, but not human, TGFBR2 rescued TGM6 antagonism in TGFBR2‐knockout cells. ITC binding measurements revealed that TGM6‐D3 bound mTGFBR2 with 40‐fold greater affinity compared to hTGFBR2. Comparing the crystal structure of TGM6‐D3:hTGFBR2 complex [19] with TGM6‐D3:mTGFBR2 complex suggested that three residues, Phe47, Ser75, and Asp141, in mTGFBR2, corresponding to Leu47, Ala75, and Glu141 in hTGFBR2 ECDs, are the primary determinants underlying the species specificity for TGM6. This was confirmed by generating mTGFBR2 and hTGFBR mutants in which these residues were swapped, with residue 47 as the primary determinant. The antagonist TGM6 binds with much higher affinity (15 nM) to mTGFBR2 than the agonists TGM1 (0.6 µM) [17] and TGM4 (116 µM) [20]. This is in line with expectations, as TGM6 needs to compete with the high‐affinity binding of TGFβ and does not possess the increased cell‐avidity TGFBR1 binding present in agonist TGMs.

Betaglycan was identified as a TGM6 co‐receptor through a biased biochemical screen, and both the orphan and ZP ECD domains in betaglycan are required for TGM6 interaction. This property is shared with TGFβ, for which the key TGFβ‐binding regions in betaglycan within the orphan and ZP domains were recently identified [30]. While TGM6‐D3 is sufficient and required for betaglycan interaction, betaglycan‐TGM6 interaction is dependent on TGFBR2. Genetic depletion of betaglycan revealed that betaglycan is a negative regulator of TGM6's antagonism of TGF‐β/SMAD signaling. In the absence of betaglycan, TGM6 binding to LRP1, the mediator of TGM6's antagonistic function, is slightly increased. The inhibitory function of betaglycan for TGM6 is in contrast to its role in TGF‐β signaling, in which betaglycan aids in presentation of TGF‐β to TGFBR and thereby potentiates TGFBR signaling [30, 31].

While TGM6‐D3 has high affinity for mTGFBR2 (15 nM, Table 1) and TGM6 competes with TGFβ for TGBR2 binding on intact cells (Figure 3C), TGM6‐D3 alone has no inhibitory effect on NIH3T3 cells [19]. Interestingly, we found that the absence of betaglycan results in TGM6 exhibiting inhibitory activity on TGF‐β signaling at high TGM6 doses. Betaglycan may help safeguard against weak TGM6 antagonism of TGFβ signaling in LRP1‐deficient cells, potentially induced by the TGM6‐D3‐TGFBR2 interaction.

LRP1 was identified as TGM6 coreceptor by affinity labeling and mass spectrometric analysis. LRP1 is a multifunctional protein that plays an essential role in endocytosis and is recycled back to the membrane surface after internalization [26]. We mapped the TGM6‐interacting domain on the LRP1 ECD to its LDLaIV cluster. TGM6‐D4/5 binds to LRP1 and occurs independently of TGFBR2 and betaglycan. Genetic misexpression studies demonstrated that LRP1 is essential for the antagonistic function and cell‐type‐specific potency of TGM6. In both NIH3T3 and NM18 cells, LRP1 is required for TGM6 to antagonize TGFβ signaling. LRP1 is expressed at much lower levels in NM18 than in NIH3T3 cells. The negative regulator of TGM6, betaglycan, is, however, expressed at much lower levels in NM18 cells than in NIH3T3 cells and may explain why TGM6 remains a potent inhibitor of TGFβ signaling in NM18 cells. The modular structure of TGM6, with its D3 and D4/5 domains that independently engage TGFBR2/betaglycan and LRP1, respectively, represents an effective means of fine‐tuning inhibitory effects across different cell types. Notably, approximately 20 years ago, LRP1 was reported to act as a TGFβ receptor and was termed the TGFβ type V receptor [32, 33, 34]. It was shown to mediate TGFβ‐induced inhibition of epithelial and myeloid cell proliferation. Other researchers have not followed up on this research.

TGM6 displaced TGFβ from interaction with TGFBR2 (and indirectly TGFBR1) on intact cells, in line with its antagonist function of TGFβ signaling [19]. TGM6‐LRP1 interaction may also indirectly contribute to TGM6 antagonism by promoting TGM6‐TGFBR2 interaction through increased cellular avidity or by sequestering TGFBR1 from TGFBR2. Notably, these ligand‐binding experiments were conducted with cells maintained on ice to prevent TGM6‐induced receptor internalization. As mentioned earlier, high doses of TGM6‐D3, in the absence of betaglycan, can inhibit TGF‐β signaling. These results also suggest that competition between TGM6 and TGFβ for TGFBR2 contributes to TGM6's antagonistic function. Importantly, we observed another mechanism by which TGM6 mediates its antagonism of TGFβ signaling. Challenging cells at 37°C with TGM6 potently decreased basal TGFBR2 expression, and the TGM6 co‐receptor LRP1 was found to mediate TGFBR2 lysosomal degradation. Blocking TGM6‐induced lysosomal degradation restored TGFBR2 levels and rescued TGFβ signaling in the presence of TGM6.

The modular structure of TGM6 may provide flexibility that allows it to interact not only with one cell type (and act in*‐cis*) but with two adjacent cells (and act in*‐trans*). However, experiments with mixed cultures of wild‐type and LRP1‐knockout cells did not support such in*‐trans* signaling between neighboring cells. In addition, TGFBR2‐expressing cells lacking LRP1 do not appear to function as a sink and do not inhibit the TGM6 effect on TGFBR^+^/LRP1^+^ cells. With LRP1 and betaglycan as co‐receptors for TGM6, the expression levels of both will determine a cell's responsiveness to TGM6‐induced antagonism of TGFβ. Cells with high LRP1 and low betaglycan will be most responsive to TGM6.

We demonstrated that by exchanging or fusing TGM domains, or by fusing TGM6‐D3 with the HER2 affibody sequence, we can create TGMs with varying cell‐specificities and control their agonistic or antagonistic functions. Of note, a fusion of TGM1 D‐3 with an engineered interleukin (IL)2 was recently found to mediate *cis*‐activation of IL‐2 and TGFβ signalling specifically in T cells that express IL‐2 receptors. This provides another example of how TGMs can be engineered to elicit cell‐type‐specific effects [35].

Furthermore, we developed a humanized TGFBR2‐VHH that, by itself, has no effect on TGFβ signaling. However, when fused to TGM1 D4/5 or to affibodies targeting EGFR or HER2, it enables cell‐specific antagonism. Mechanistically, TGFBR2‐based BsAbs and TGM6 inhibit TGFβ signaling in part differently: TGFBR2 VHH‐based BsAbs and TGM6 inhibit TGFβ‐TGFBR2 binding in co‐receptor expressing cells, while TGM6 uniquely triggers TGFBR2 degradation in LRP1 coreceptor expressing cells. Of note, unlike TGMs (and derivatives), TGFBR2‐VHH‐based BsAbs lack parasitic sequences and efficiently target human cells.

The ability of TGFBR2‐VHH‐based BsAbs to inhibit TGFβ signaling is distinct from TGFBR2 ECD‐Fc‐based ligand traps. The latter are engineered fusion proteins that block TGFβ from binding to TGFBR2, either alone or fused to other targeting moieties, such as Programmed cell death protein ligand 1 (PD‐L1) and Cluster of Differentiation 4 (CD4) [36, 37]. While fusion with other targeting modalities may prompt them to act more locally. However, unlike the TGFBR2‐based BsAbs, the ligand traps do not block TGFβ signaling in a cell‐selective manner and lead to lower systemic TGFβ levels, potentially causing adverse effects. For example, in clinical studies of a PD‐L1 antibody fused to TGFBR2‐ECD, patients experienced a reduction in circulating TGFβ1 levels of more than 97% within two weeks of treatment initiation, which remained reduced up to 12 weeks [38].

Ongoing efforts in our laboratory focus on further optimizing TGFBR2‐VHH to enhance its inhibitory potency against TGFβ signaling within a BsAb framework while maintaining its cell‐type‐specific activity. Achieving this selectivity is significantly more complex for cell‐type‐specific antagonists than for targeted agonists; the latter merely require co‐receptor‐mediated potentiation of the low‐affinity TGFBR1 and TGFBR2 binding to mediate cell‐type‐specific avidity.

## 4 Conclusion

The mechanism‐based studies on TGMs inspired the creation of a versatile set of designer agents to precisely manipulate TGFβ signaling, tailored to specific cell type(s). Moreover, the innovative TGFBR2‐VHH‐based BsAbs could serve as a foundation for developing new drugs that target cancer and other human diseases associated with dysregulated TGFβ activity in a cell‐selective manner, potentially avoiding the on‐target side effects of existing TGFβ‐targeting agents.

## 5 Experimental Section

### 5.1 Materials

Cycloheximide (01810) and hydroxychloroquine (H0915) were obtained from Sigma–Aldrich; bafilomycin A1 (10‐2060) was obtained from Focus Biomolecules; and bortezomib (SC‐217785) was obtained from Santa Cruz. Recombinant human TGF‐β3, expressed in *E. coli* as the mature C‐terminal signaling domain, was refolded and purified by a patented method [39].

### 5.2 Cell Lines

The following cell lines, 293T, NIH3T3, NM18 (a subline of the mouse mammary gland epithelial cell line NMuMG) [22], MDA‐MB‐231, B16F10, L6E9, HT‐1080, MFB‐F11, and HepG2 were maintained in DMEM (41966‐052, Gibco), and U87‐MG in EMEM (670086, Gibco), both supplemented with 10% fetal bovine serum (FBS) (S1810‐500, Biowest) and penicillin/streptomycin. Generation of NIH‐3T3 fibroblasts (CRL‐1658) containing the fluorescent‐based TGF‐β/SMAD3 transcriptional reporter, i.e., CAGA‐MLP‐dynGFP, was previously described [40]. Cells were routinely tested for mycoplasma absence, and human cell lines were authenticated by Short Tandem Repeat (STR) profiling.

### 5.3 Selection, Affinity Maturation, and Characterization of TGFBR2 VHH

TGFBR2‐specific VHHs were generated by Hybrigenics (Évry‐Courcouronnes, France). A synthetic VHH library containing 3.109 clones was screened using HEK293T expressing TGFBR2. After three rounds of selection, 90 VHHs were selected and analyzed by Phage FACS for binding to TGFBR2; 28 were positive. After sequencing and a confirmation analysis, we established 14 independent VHHs. The highest‐affinity binder from our initial selection, i.e., clone (cl.) 6, was subsequently used for in vitro affinity maturation. To this end, a mutant TGFBR2‐VHH library (2–5 × 10^6^) derived from the parental clone sequence was generated by introducing mutations restricted to the complementarity‐determining regions (CDRs). Thereafter, the yeast‐expressing mutant library was subjected to three rounds of sorting using recombinant, carboxy‐terminally Avi‐tagged TGFBR2 ECD as bait. The concentration of the TGFBR2 ECD domain was gradually decreased to select nanobodies with the highest binding affinities. Highest‐affinity binders were characterized for the efficiency of TGFBR2 immunoprecipitation and FACS assays. All clones were DNA sequenced, and high‐affinity clones (i.e., cl.6A to cl.6D) or mutational variants of different clones (i.e., cl.6‐Y62S) were used in subsequent biological experiments. Sequences of the cl.6 and its derivatives are listed in Table S2. Other clone sequences are available upon request.

### 5.4 DNA Constructs

#### 5.4.1 LRP1 Constructs

Full‐length mLRP1 (non‐tagged in pcDNA3) was a gift from Anton J. M. Roebroek, KU Leuven, Belgium [41]. A FLAG tag, along with an NheI site, was inserted between amino acids 23 and 24. PCR was used to generate deletion constructs in the pcDNA3 vector. The LDLaII and LDLaIV clusters were made by PCR and cloned into the pDISPLAY vector to create a membrane‐bound protein. For stable expression, D4 and LDLaIV were cloned into a lentiviral vector (pLV‐CMV‐IRES‐NEO). Cluster LDLaIV was also cloned from the pDISPLAY vector in pcDNA3 without the transmembrane domain (but with the signal peptide) to create a secreted protein. Full‐length hLRP1 (without signal peptide and tag) in pCR8 (gateway donor vector) was a gift from Piet Gros, Utrecht University, the Netherlands [42]. Signal peptide was synthesized, and a full‐length human LRP1 was constructed in pcDNA3. This construct was used to generate the D4 deletion mutant by PCR (in pLV‐CMV‐IRES‐NEO). The full‐length human LRP1 was used as a template to create a pDISPLAY construct with cluster LDLaIV (to make a membrane‐bound protein. As with the mouse LDLaIV cluster, it was subcloned into pcDNA3, lacking the transmembrane domain (but retaining the signal peptide), to generate a secreted protein.

#### 5.4.2 rBetaglycan (TGFBR3) Constructs

rBetaglycan (TGFBR3) WT, delta Orphan, and delta ZP expression constructs were a gift from Fernando López‐Casillas, Universidad Nacional Autónoma de México (UNAM), México City, México [43]. The constructs were re‐cloned from the pCMV5 vector into a lentiviral vector (pLV‐CMV‐IRES‐NEO).

#### 5.4.3 TGFBR2 Constructs

hTGFBR2 and mTGFBR2 were cloned into the pENTR1A vector. For mTGFBR2, a construct was generated in which four silent mutations were introduced in the target site of guide 2 to prevent it from being targeted. The GATEWAY system (Invitrogen) doxycycline‐inducible lentiviral constructs were generated in the pCW57.1 vector, a gift from David Root (Addgene plasmid # 41393; http://n2t.net/addgene:41393; RRID:Addgene_41393

#### 5.4.4 TGFBR2‐VHH Expression Constructs

TGFBR2‐VHH TGM1‐D4‐5, TGFBR2‐VHH‐Fc, zHER2‐TGFBR2‐VHH, and zEGFR‐TGFBR2‐VHH were made by cloning synthetic constructs into the pFUSE vector (Invivogen). For amino acid sequences, see Table S2.

#### 5.4.5 HER2 Expression Construct

Human HER2 was cloned into the pLV‐CMV‐IRES‐PURO by PCR from a HER2 cDNA clone (ccsbBroadEn_14631, Broad Institute).

#### 5.4.6 Expression Constructs for TGM1 and TGM6 (Fusion) Proteins

Expression of *H. polygyrus* TGM1 and TGM6 (deletion) was conducted in HEK293 cells, using His‐tagged proteins and metal‐chelating affinity chromatography. Construction and purification methods are as previously described [8, 9]. TGM6‐D3/TGM1‐D45 and TGM1‐D12/TGM6 fusion constructs were generated by three‐point ligation using two digested PCR products. The fragments were cloned into the SfiI and NotI sites of expression vector pSECTAG2a (Thermo Fisher Scientific). The TGM6‐mut was generated by introducing mutations that replace Arg38, Ile78, and Tyr93 with alanine. Constructs were ordered from GeneArt, and *Asc*I and *Not*I restriction sites were used for insertion into pSecTag2A vector, and thereafter TGFM6‐mut was expressed in HEK293 and purified as for other TGM6 proteins.

### 5.5 Expression and Purification of TGM6‐D3/TGM1‐D45 and TGM1‐D12/TGM6 Fusions

293T cells were transfected using PEI‐MAX (Polysciences) with expression constructs for TGM6‐D3/TGM1‐D45, TGM1‐D12/TGM6, and TGFBR2‐VHH‐based fusions using PEI‐MAX (24765, Polysciences). See Table S3 for information on the amino acid sequences of TGM6‐D3/TGM1‐D45m and TGM1‐D12/TGM6 fusion constructs. After 24 h, the cells were washed 1x with phosphate‐buffered saline (PBS), and fresh serum‐free medium was added overnight. Next, the conditioned media were harvested, filtered through a 0.2 µm Puradisc FP 30 mm filter (Whatman), and used in the experiments. For vehicle control, conditioned medium from empty‐vector*‐trans*fected cells was used. To compare TGM6‐D3/TGM1‐D45 with TGM6, TGM6 was also expressed in parallel and processed in the same manner.

### 5.6 Expression and Purification of zHER2‐Link‐TGM6‐D3

zHER2‐link‐TGM6‐D3 was produced using the ALiCE system (AL00000003, LenioBio) using their pALiCE1 vector, all according to their manual. The proteins were purified using Strep‐Tactin 4Flow beads (17577466, IBA Lifesciences) according to the manufacturer's instructions. See Table S3 for information on the amino acid sequence of zHER2‐link‐TGM6‐D3. The amino acid sequence and functional properties of zHER2‐affibody were previously described [28].

### 5.7 Expression and Purification of TGM6‐D3 and TGFBR2 for ITC and Crystallization

The amino acid sequences of the TGM6‐D3, hTGFBR2, and mTGFBR2 proteins used in this study are presented in Table S4. TGM6‐D3 and hTGFBR2 were each produced as insoluble inclusion bodies in E. coli BL21 (DE3), refolded, and purified by high‐resolution ion‐exchange chromatography (Source S and Source Q, respectively; Cytiva, Piscataway, NJ), as previously described [19, 44]. mTGFBR2 was produced and purified similarly to hTGFBR2, the only difference being that the final high‐resolution ion‐exchange purification using a Source Q column (Cytiva, Piscataway, NJ) was performed in 25 mM Tris‐HCl, pH 8.0, instead of 25 mM MES‐HCl, pH 6.0. The purity and identity of each protein were verified using non‐reducing SDS‐PAGE and by measuring the intact mass using liquid chromatography electrospray ionization time‐of‐flight mass spectroscopy (Micro TOF, Bruker Daltonics, Billerica, MA).

### 5.8 Isothermal Titration Calorimetry

ITC data were generated using a Microcal PEAQ‐ITC instrument running version 1.40 of the Malvern PEAQ‐ITC control software (Malvern Instruments, Westborough, MA). All experiments were performed in 25 mM HEPES, 150 mM NaCl, 0.05% NaN_3_, pH 7.4. Concentrations of the proteins in the syringe and sample cell are listed in Table 1. Before each experiment, all proteins were dialyzed into ITC buffer, then diluted as necessary before being loaded into the sample cell or syringe. For each titration, either 19 injections of 2.0 µL or 25 injections of 1.5 µL were performed, with an injection duration of 4 s and a spacing of 150 s; two independent data sets were collected for each titration. Integration and data fitting were performed using *Nitpic* 2.1.0 [45] and *Sedphat 15.2b* [23, 24] with outliers removed as necessary. Each binding experiment, comprised of two independently measured titrations, was globally fit to a one‐to‐one binding model. The error estimates were derived using *SEDPHAT 15.2b* [23, 24], and the data were plotted using *GUSSI 2.1.0* [46].

### 5.9 Crystallization, Integration, Reduction, and Phasing

Crystallization was performed at 16 °C using the sitting‐drop method in 96‐well plates with 50 µL of well solution. The setup of crystallization screens was performed using a Mosquito robot (SPT Labtech, Hertfordshire, U.K.) by mixing 100 nL of protein complex with 100 nL of well solution. When looped, the crystals were transferred to a drop of well‐solution adjusted with cryoprotectant. Each crystal was mounted in a nylon loop. Excess well solution was wicked off, and the looped crystals were flash‐frozen in liquid nitrogen before being shipped at liquid nitrogen temperature for remote data collection. The TGM6‐D3:mTGFBR2 complex crystal was crystallized with 35 mg mL^−1^ protein complex and a well‐solution of 0.1 M sodium HEPES pH 7.5, 10% PEG 4000, and 0.1 M MgCl_2_. The cryoprotectant was 25% glycerol. Diffraction data were collected at the Brookhaven National Laboratory National Synchrotron Light Source II (NSLS‐II, beamline 17‐ID‐1). Diffraction images were integrated with iMosflm [47], and the space group of each crystal was confirmed with Pointless [48, 49]. The diffraction data were reduced with Aimless [50], Ctruncate [51], and the Uniquify script [52] in the CCP4 software suite. Phaser [53] was used for molecular replacement, with the TGM6‐D3:hTGFBR2 complex structure as the search model [19]. Several cycles of refinement using *phenix.refine* [54] and model building using COOT [55, 56] were performed to determine the final structure. Data collection and refinement statistics are shown in the crystallography data table (Table S1). Images were generated using the open‐source PyMOL, with density thresholds of 1.5 and 1.0, and root‐mean‐square deviation (RMSD) values of 0.5 and 0.4 for the human and mouse TGFBR2 complexes, respectively.

### 5.10 Statistical Analysis

Statistical analyses were performed using an unpaired, two‐sided t‐test with unequal variances in Excel. *P* *<* 0.05 was considered statistically significant. *0.01 < *P* < 0.05; ***0.001 < *P* < 0.01; ***0.0001 < *P* < 0.001; ****P < 0.0001.

## Author Contributions

PtD, APH, RMM, and MvD conceived and designed the study. MvD, TS, KF, JZ, GvdZ, LP, CH, CC, AM, RG‐P, and PvV designed and performed the experiments. MvD, TS, JZ, KF, GvdZ, LP, CH, AM, R G‐P, PvV, RMM, APH, and PtD analyzed the data. PtD coordinated the study and wrote the paper with input from all authors. RMM, PtD, JZ, KF, and APH provided funding.

## Funding

This work was funded by grants awarded from the Oncode Institute base fund and NWO (Grant ID: https://doi.org/10.61686/AHJBX34229) to PtD, Chinese Scholarship Council (to JZ and KF), a Wellcome Trust Discovery award to RMM, APH, and PtD, Wellcome Trust grant (306173) to RM, and NIH R03 (AI53915) and NIH F30 (AI157069) grants to APH and AM, respectively.

## Ethics Statement

No clinical samples were used, nor were animal studies performed.

## Conflicts of Interest

Peter ten Dijke, Maarten van Dinther, and Andrew Hinck filed a patent on cell‐type‐specific inhibitors of TGFBR2 signaling. The other authors declare no competing interests.

## Supporting information




**Supporting File**: advs75322‐sup‐0001‐SuppMat.docx.

## Data Availability

All data needed to evaluate the conclusions in the paper are present in the paper and/or the Supplementary Materials. The structural data for the TGM6‐D3:mTGFBR2 ECD complex were deposited in the PDB under accession code 9YJ4. The mass spectrometry proteomics data have been deposited to the ProteomeXchange Consortium via the PRIDE partner repository [57] with the dataset identifier PXD065047.
