# Supplementary material for: Decoding the Mechanism of Action of a Parasite TGFβ Antagonist Inspires the Creation of Cell‐Type‐Specific TGFβ Modulators
Source: Adv Sci (Weinh). 2026 Apr 20;13(38):e75322. doi: 10.1002/advs.75322 (PMC13335435; doi:10.1002/advs.75322)
Supplement: Supplementary file 1 — Supporting File: advs75322‐sup‐0001‐SuppMat.docx. [file ADVS-13-e75322-s001.docx]

Supporting Information

**Decoding the Mechanism of Action of a Parasite TGFβ antagonist Inspires the Creation of Cell-type-specific TGFβ Modulators**

*Maarten van Dinther^1^, Tristin Schwartze^2^, Jiying Zhang^1^, Kun Fan^1^, Gerard van der Zon^1^, Luke Power^3,7^, Cynthia Hinck^2^, Claire Ciancia^3,8^, Ananya Mukundan^2^, Roman Gonzalez-Prieto^4,5^, Peter van Veelen^6^, Rick M. Maizels^3#^, Andrew P. Hinck^2#^, and Peter ten Dijke^1#*^*

This PDF file includes:

Additional Methods

Figures S1 to Figure S11

Table S1 to Table S6

**Additional Methods**

**1.1. Guide RNA’s**

Two different knockout guides against each target were designed using the ChopChop web tool (https://chopchop.cbu.uib.no/). The guides were cloned into the pLENTI-CRISPR-V2 plasmid (a gift from Feng Zhang; Addgene plasmid # 52961) [1]. See Table S5 for guide sequences.

- 1. **Lentiviral cell transduction and selection**

To generate stable cell lines, cells were transduced with a lentivirus produced in 293T cells. In brief, 293T cells were transfected (using PEI MAX® (24765, Polysciences)) with helper plasmids and the lentiviral plasmid, and on the following day, the transfection media were refreshed. On the second day, the conditioned media were collected and filtered to obtain a cell-free solution. Next, cells were incubated overnight with the filtered conditioned medium; 48 hours later, cells were selected with an appropriate antibiotic. After knockout selection, the knockout cells were used either as a pool or to generate single-cell clones.

- 1. **Analysis of mRNA expression in cell lines.**

We analysed the mRNA expression in cancer cell lines using the Human Protein Atlas database (https://www.proteinatlas.org/) [2].

- 1. **Transcriptional fluorescent protein-based reporter assay**

To measure SMAD3 transcriptional activity, we used a fluorescence-based transcriptional reporter in which concatemerized SMAD3/4 binding elements (CAGA sequences) were cloned upstream of a minimal promoter. SMAD3/4 binding elements were derived from TGFβ/SMAD-responsive gene *SERPIN1*, encoding plasminogen activator protein 1 [3]. Cells containing either the CAGA-dynamic green fluorescent protein (dynGFP) or the CAGA-mCHERRYd2 reporter were seeded in 96-well plates. The next day, cells were pretreated with TGM6 for 30 minutes, then stimulated with TGFβ (in complete medium), and placed in the IncuCyte S3 live-cell imaging system (Sartorius). The cells were imaged after an overnight (21-hour) treatment. Fluorescence intensity was analyzed using the IncuCyte software. TGF-β receptor/SMAD3 signaling was measured with an MFB-F11 cell assay as previously described [4]. All experiments were performed at least three times (and/or conducted on multiple independent cell clones), and representative results are shown. In experiments where the effect of TGM (derivative) or BsAb on TGFβ-induced transcriptional response was measured, TGM (derivative) or BsAb was added 30 minutes prior to TGFβ (overnight treatment).

- 1. **Actin stress fiber staining**

NM18 cells were grown in a 96-well dish at 20% confluence. Cells were pre-treated with 100 ng/ml TGM6 for 30 minutes, after which the cells were stimulated with 1 ng/ml TGFβ for 48 hours. Cells were fixed with 4% paraformaldehyde (PFA) after 2 days, permeabilized with 0.1% Triton in PBS, blocked with 3% bovine serum albumin (BSA) in PBS, incubated with phalloidin-ALEXA-488 (Molecular Probes, A12379) for 1 hour, and then washed. The cells were imaged using the IncuCyte S3 (Sartorius). All experiments were performed at least three times, and representative results are shown.

- 1. **Iodination and crosslinking.**

Purified recombinant proteins were labelled by iodination, biotinylation. Iodination of TGM6 or TGFβ proteins was performed by the chloramine T method, and cells were subsequently affinity-labeled with the radioactive ligand as previously described [5,6]. In short, cells were incubated with the radioactive protein on ice for 3 hours. After incubation, the cells were washed, and crosslinking was performed using 0.27 µM disuccinimidyl suberate (DSS, Pierce, 21555) and 0.07 µM bis(sulfosuccinimidyl) suberate (BS3, Pierce, 21580) for 15 minutes. Cells were washed, scraped, and lysed. Lysates were incubated with antibodies overnight (4°C) and were precipitated using protein A Sepharose (Amersham, 17-0963-03). Samples were washed, boiled in SDS sample buffer, and subjected to SDS-PAGE. Gels were dried, exposed to a phosphor screen (FUJIFILM, BAS-SR2040), and then imaged using the Typhoon (Amersham). Antibodies used for immunoprecipitation are homemade in ten Dijke laboratory and have previously been described [6-9]. In the competition experiments, TGM (derivative) or BsAb was added 30 minutes prior to iodinated TGFβ.

- 1. **Western Blotting and detection**

Cells were rinsed with ice-cold PBS before being lysed in lysis buffer. Protein concentrations were measured using the DC protein assay kit (Bio-Rad, 5000116), and samples were adjusted accordingly. After adding sample buffer and boiling for 5 minutes, the samples were separated on 8% SDS-polyacrylamide gels or on precast gradient gels (NuPAGE 4-12% gel, Invitrogen, WG1403BOX) and subsequently transferred to a nitrocellulose membrane (Protran, Amersham, 10600003). Transfer efficiency was assessed using Ponceau S. Membranes were blocked in TBS-T containing 5% skim milk for 1 hour at room temperature. Primary antibodies were incubated overnight at 4 °C. Secondary antibodies were incubated for 1 h at room temperature before detection using ECL (Clarity Western ECL Substrate, Bio-Rad, 170-5061) and the Chemidoc Imaging System (Bio-Rad). Band intensities were analyzed using Image Lab software (Bio-Rad). We used glyceraldehyde-3-phosphate dehydrogenase (GAPDH) expression or Ponceau S staining of the transferred protein as an equal loading control. See Table S6 for a list of the primary and secondary antibodies used in this study for Western blot analysis. All experiments were performed at least three times (and/or conducted on multiple independent cell clones), and results from representative experiments are shown. In experiments where the effect of TGM (derivative) or BsAb on TGFβ-induced pSMAD2 response was measured, TGM (derivative) or BsAb was added 30 minutes prior to TGFβ (60 minutes) addition (except in cases stated otherwise).

- 1. **Biotinylation and pulldown**

Ten µg of TGM6 was incubated with EZ-Link™ Sulfo-NHS-LC-Biotin (Pierce, 21335) for 30 minutes at RT. The reaction was stopped by the addition of 50 mM Tris-HCl (pH 7.4). The reaction was purified using a G-25 column (GE Health, 28922529). For the pulldown, cells were washed with PBS and then incubated with biotinylated protein(s) for 3 hours on ice. After incubation, the plates were washed 3 times with PBS (Fresenius Kabi), and the cells were harvested in 1x Cell Lysis Buffer (Cell Signaling Technologies, 9803). After spinning, the supernatant was incubated with Neutravidin beads (Pierce, 29201) for 1 hour at 4°C (rotating). Beads were washed 4 times with lysis buffer and 3 times with 50 mM ammonium bicarbonate (Sigma-Aldrich, 09830), using fresh LoBind tubes for each wash (Eppendorf, 0030 108.116). The beads were resuspended in 250 µl of 50 mM ammonium bicarbonate with 250 ng of mass spec grade trypsin (Promega V5113), incubated overnight at 37°C (with agitation), after which peptides were recovered from the beads with a pre-washed 0.4 µm filter (Ultrafree MC HV, Millipore, UFC30HV00) and subjected to mass spectrometry analysis.

- 1. **Alphafold**

The predicted structure of TGM6 with mTGFBR2 ECD and LRP1 mLRP1-LDLaIV

was generated using AlphaFold3 (https://alphafoldserver.com/) [10].

- 1. **Mass spectrometry**

Peptides were desalted using StageTips [11] and analyzed on an Orbitrap Fusion LUMOS (Thermo Fisher Scientific) hybrid mass spectrometer coupled to an EASY-nLC 1200 system (Proxeon, Odense, Denmark). Three technical repeats were performed, injecting 2%, 10%, and 50% of the sample, respectively. Digested peptides were separated using a 50 cm long fused silica emitter (FS360-75-15-N-5-C50, New Objective, Massachusetts, US) in-house packed with 1.9 μm C18-AQ beads (Reprospher-DE, Pur, Dr. Maisch, Ammerbuch-Entringen, Germany) and heated to 50°C in a Column Oven for ESI/Nano Spray (Sonation, Germany). Peptides were separated by liquid chromatography using a gradient from 2% to 32% acetonitrile with 0.1% formic acid for 30 minutes, followed by column reconditioning for 22 minutes. A Lock Mass of 445.12003 (polysiloxane) was used for internal calibration. Data were acquired in Data-Dependent Acquisition (DDA) mode with a TopSpeed method, with a cycle time of 3 s, a scan range of 400-1500 m/z, and resolutions of 120,000 and 30,000 for MS1 and MS2, respectively, using the Orbitrap as detector in both cases. For MS2, an isolation window of 1.2 m/z and an HCD collision energy of 32% was applied. Precursors with a charge of 1 and higher than 6 were excluded from triggering MS2, as well as previously analyzed precursors with a dynamic exclusion window of 10s.

- 1. **Mass spectrometry data analysis**

Mass spectrometry data were analyzed using MaxQuant v1.6.14.0 [12] with the following modifications: the maximum number of missed cleavages by trypsin/p was set to 4. Searches were performed against an in silico-digested database of the mouse proteome, including isoforms and canonical proteins (Uniprot, 22nd April 2021). Oxidation (M), Acetyl (Protein N-canonical proteins (Uniprot, 22nd April 2021). Oxidation (M), Acetyl (Protein N- term), were set as variable modifications with a maximum of 3. Carbamidomethyl (C) was disabled as a fixed modification. Label-free quantification was activated, not enabling Fast LFQ. The match-between-runs feature was activated with the default parameters.

- 1. **Scientific figure creation**

Several figures from the NIAID Visual & Medical Arts (10/7/2024, NIAID NIH BioArt, (bioart.niaid.nih.gov)) and three figures from scidraw (scidraw.io, CC-BY 4.0) were used in the generation of the schematic figures 3A and 4A. Figures used from NIH bioart are: Receptor (BIOART-000429), Receptor protein (BIOART-000438), Generic Mass Spec Machine (BIOART-000639), Liquid Chromatography Machine (BIOART-000638), and Mass Spectrometry Graph (BIOART-000582). Figures used from scidraw; Eppendorf tube closed and Petri Dish by Diogo Losch De Oliveira, and Progenitors by Roberta Schellino.

**References:**

1. N. E. Sanjana, O. Shalem, F. Zhang, Improved vectors and genome-wide libraries for CRISPR screening. *Nat. Methods* **11**, 783–784 (2014).
2. H. Jin, C. Zhang, M. Zwahlen, et al., Systematic transcriptional analysis of human cell lines for gene expression landscape and tumor representation. *Nat. Commun.* **14**, 5417 (2023).
3. D. L. Marvin, L. You, L. Bornes, et al., Dynamic Visualization of TGF-β/SMAD3 Transcriptional Responses in Single Living Cells. *Cancers* **14**, 2508 (2022).
4. M. van Dinther, K. T. Cunningham, S. P. Singh, et al., CD44 acts as a coreceptor for cell-specific enhancement of signaling and regulatory T cell induction by TGM1, a parasite TGF-β mimic. *Proc. Natl. Acad. Sci. U.S.A.* **120**, e2302370120 (2023).
5. C. A. Frolik, L. M. Wakefield, D. M. Smith, M. B. Sporn, Characterization of a membrane receptor for transforming growth factor-β in normal rat kidney fibroblasts. *J. Biol. Chem.* **259**, 10995–11000 (1984).
6. H. Yamashita, P. ten Dijke, D. Huylebroeck, et al., Osteogenic protein-1 binds to activin type II receptors and induces certain activin-like effects. *J. Cell Biol.* **130**, 217–226 (1995).
7. P. ten Dijke, H. Yamashita, H. Ichijo, et al., Characterization of type I receptors for transforming growth factor-β and activin. *Science* **264**, 101–104 (1994).
8. P. ten Dijke, H. Yamashita, T. K. Sampath, et al., Identification of type I receptors for osteogenic protein-1 and bone morphogenetic protein-4. *J. Biol. Chem.* **269**, 16985–16988 (1994).
9. H. Yamashita, H. Ichijo, S. Grimsby, A. Morén, P. ten Dijke, K. Miyazono, Endoglin forms a heteromeric complex with the signaling receptors for transforming growth factor-β. *J. Biol. Chem.* **269**, 1995–2001 (1994).
10. J. Abramson, J. Adler, J. Dunger, et al., Jumper, Accurate structure prediction of biomolecular interactions with AlphaFold 3. *Nature* **630**, 493–500 (2024).
11. J. Rappsilber, M. Mann, Y. Ishihama, Protocol for micro-purification, enrichment, pre-fractionation and storage of peptides for proteomics using StageTips. *Nat. Protoc.* **2**, 1896–1906 (2007).
12. S. Tyanova, T. Temu, J. Cox, The MaxQuant computational platform for mass spectrometry-based shotgun proteomics. *Nat. Protoc.* **11**, 2301–2319 (2016).
13. P. J. Hart, S. Deep, A. B. Taylor, Z. Shu, C. S. Hinck, A. P. Hinck, Crystal structure of the human TbetaR2 ectodomain--TGF-β3 complex. *Nat. Struct. Biol.* **9**, 203–208 (2002).


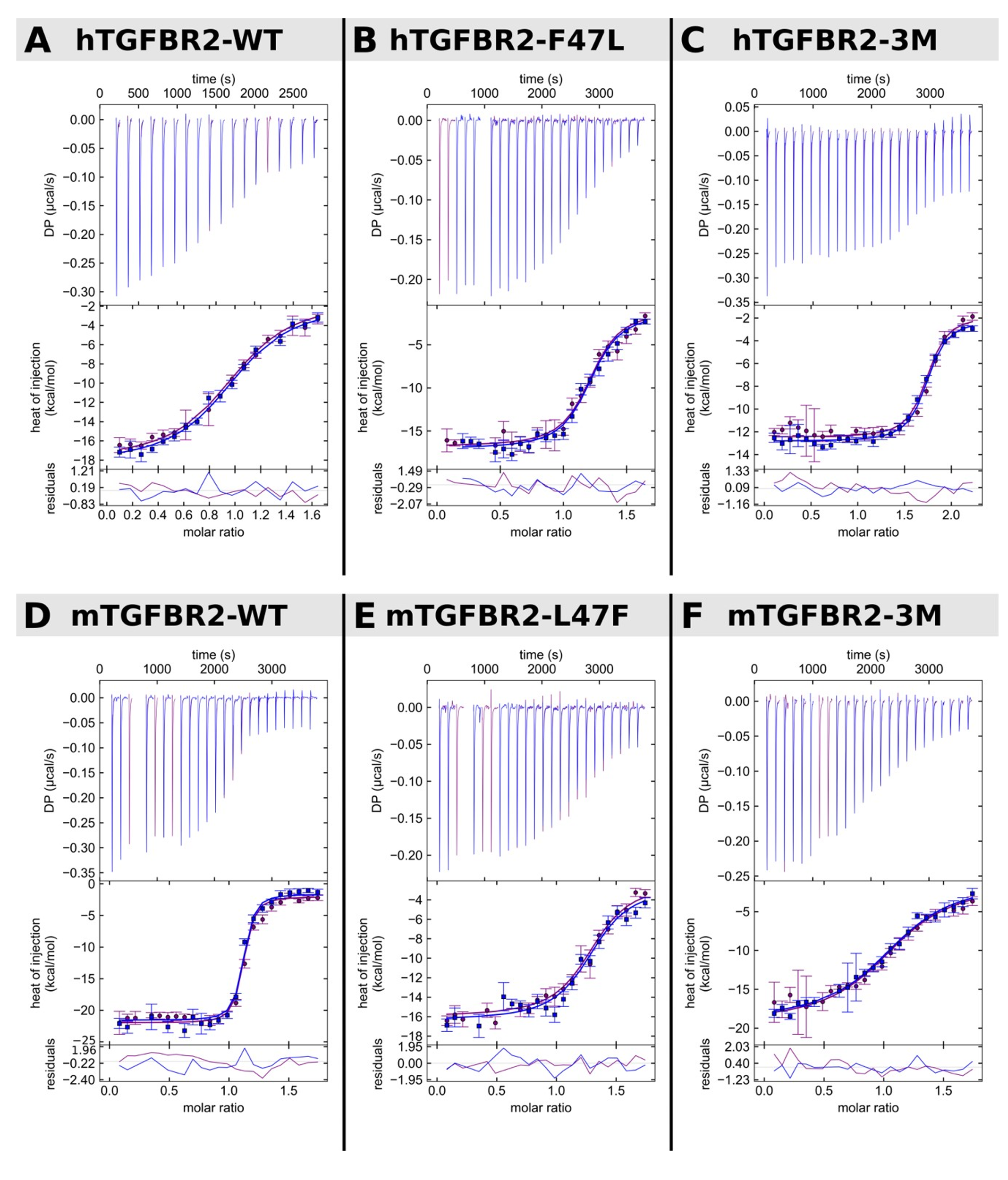


**Figure S1. Isothermal titration calorimetry (ITC) data for binding of TGM6-D3 to wild-type mouse and human TGFBR2 ECD and variants**. (**A-F**) ITC binding data between TGM6-D3 in the cell and TGFBR2 in the syringe. Wild-type human TGFBR2 and the F47L and F47L, S75A, D141E (3M) variants are shown in panels **A-C**, respectively, while wild-type mouse TGFBR2 and the L47F and L47F, A75S, and E141D (3M) variants are shown in panels **D-F**, respectively. Each ITC sub-panel comprises thermograms (upper panel), mean integrated heats (middle panel), and fit residuals (lower panel). Duplicate datasets were collected (purple and blue), and the data from both were globally fit to a 1:1 model to obtain a single KD, DH, and incompetent fraction in the cell and syringe (shown by continuous lines in the middle panel). Fitted values are provided in Table 1.


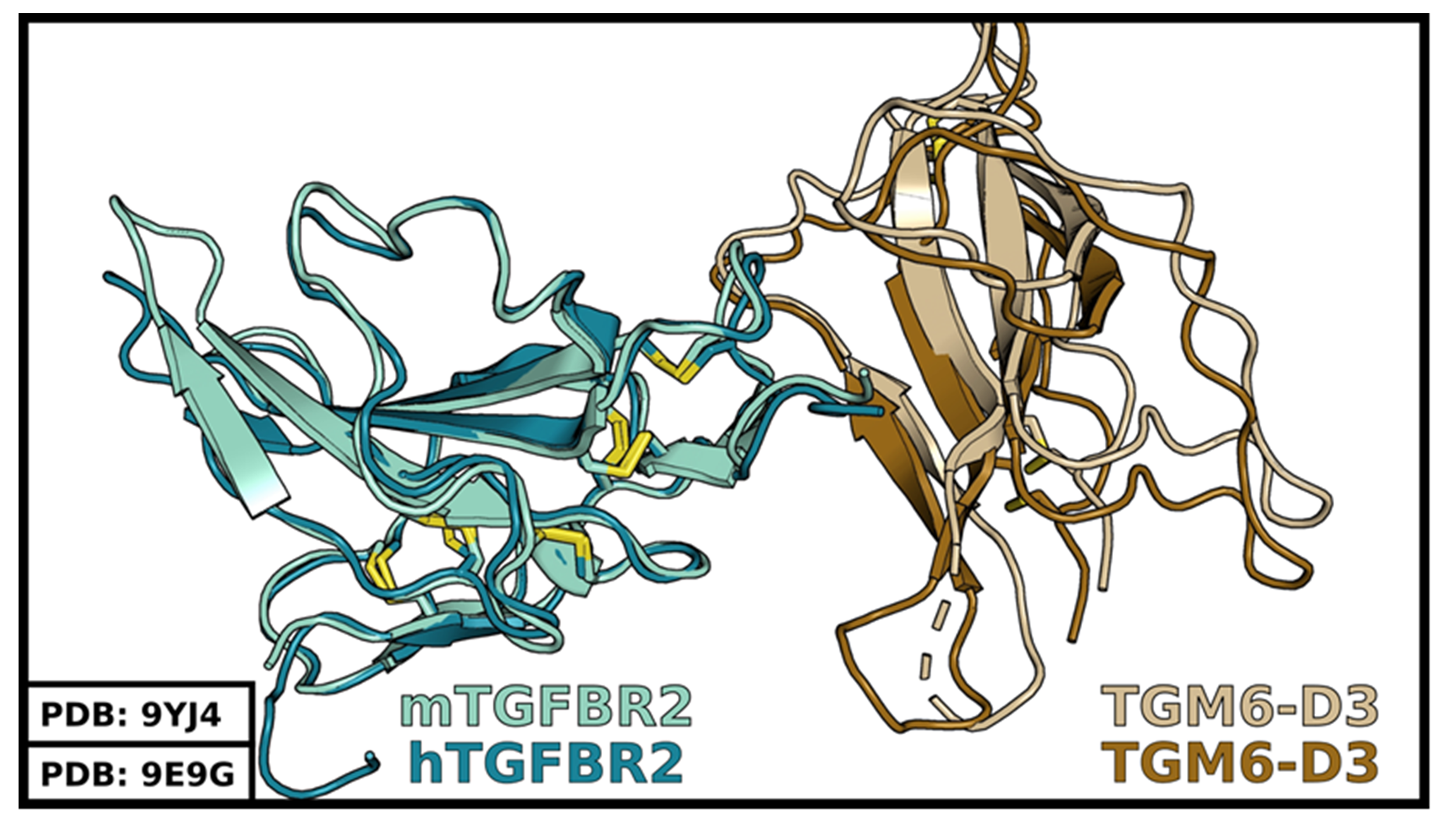


**Figure S2. Overall Structure of TGM6-D3 in complex with hTGFBR2 or mTGFBR2.** The asymmetric unit of TGM6-D3 in complex with hTGFBR2 contains a single copy of the complex. Overlay of mTGFBR2 (light green):TGM6-D3 (light brown) and hTGFBR2 (dark green):TGM6-D3 (dark brown).


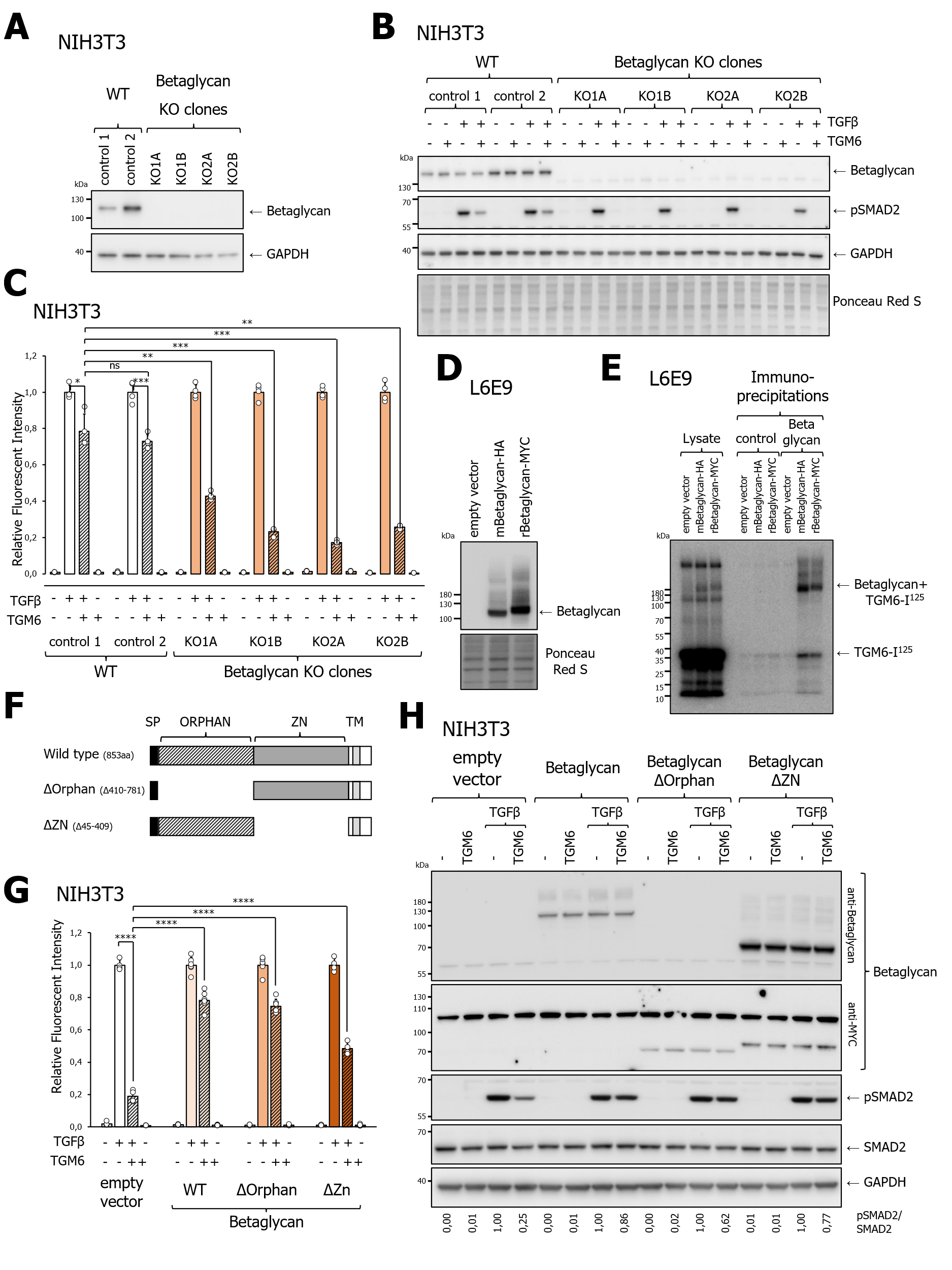


**Figure S3. Betaglycan is a negative regulator of TGM6-mediated antagonism of TGFβ/SMAD signaling response, and the Orphan and Zona Pellucida domains of Betaglycan are both functionally required.** (**A**) Analysis of betaglycan expression in wild-type (control 1 and 2) and knock-out clones (KO1, KO2, KO3, and KO4) as measured by Western blot analysis. (**B** and **C**) Effect of betaglycan knockout on antagonism by TGM6 on TGFβ-induced SMAD2 phosphorylation (**B**) and TGFβ/SMAD3 transcriptional reporter response in NIH3T3 cells (**C**). (**D**) Analysis of ectopic expression of mouse and rat betaglycan in L6E9 myoblasts. Mouse or rat betaglycan was transiently expressed in L6E9 cells. (**E**) Betaglycan-transfected L6E9 cells (characterized in **D**) were used to assess TGM6 binding to mouse or rat betaglycan. Iodinated TGM6 was used to affinity-label cell-surface proteins; betaglycan was immunoprecipitated from cell lysates, and signals were analyzed by autoradiography. (**F**) Schematic representation of two betaglycan deletion constructs. (**G** and **H**) Effect of wild-type betaglycan or betaglycan deletion mutants in NIH3T3 cells s on TGFβ/SMAD3-induced dynGFP transcriptional response (**G**) and SMAD2 phosphorylation (**H**). pSMAD2/SMAD2 ratios are indicated underneath GAPDH blot results in **H**. TGM6 (100 ng/ml) was added 30 minutes before the addition of TGFβ (1 ng/ml). **S3B** and **S3C** correspond to the extended dataset for Figs. **3D-E** and **3F**, respectively.


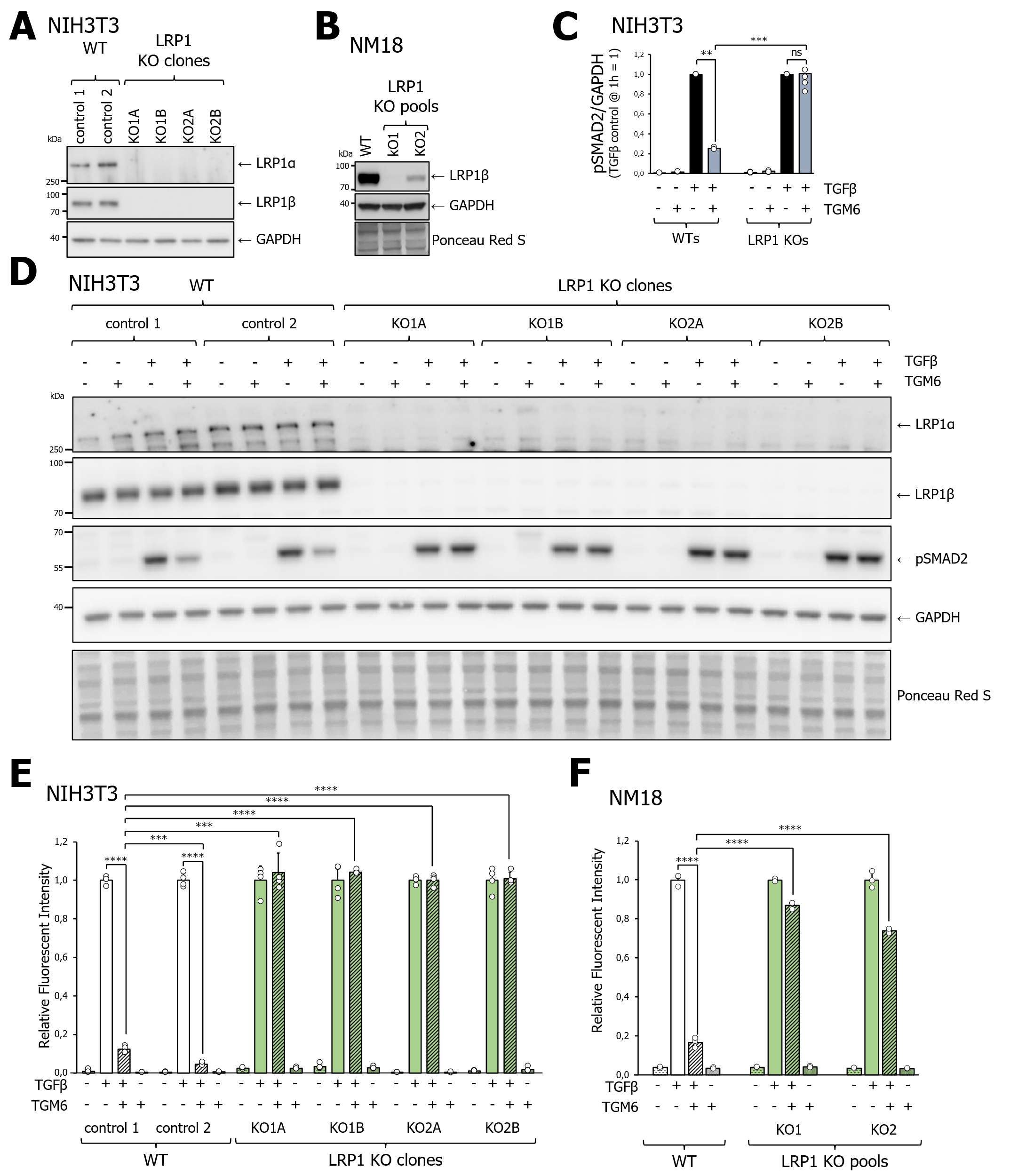


**Figure S4. LRP1 is a co-receptor for TGM6.** (**A** and **B**) Validation of LRP1 knock-out in NIH3T3 and NM18 cells. (**A**) Expression of LRP1 (sub-units LRP1α and LRP1β) as measured by Western blot analysis in NIH3T3 wild-type clones (control 1 and control 2) and LRP1 KO clones (KO1A, KO1B, KO2A, and KO2B). KO1 and KO2 were made using different guide RNAs. (**B**) Expression analysis of LRP1β in LRP1 wild-type cells and LRP1 KO pools (KO1 and KO2) as measured by Western blot analysis. (**C** and **D**) Effect of LRP1 deficiency in NIH3T3 cells on antagonism of TGM6 on TGFβ/SMAD2 phosphorylation response. **C** shows the quantification of pSMAD2/GAPDH ratios of Western blot data in **D**. (**E** and **F**) Effect of LRP1 deficiency in NIH3T3 cells on the antagonism of TGM6 on TGFβ/SMAD3 transcriptional activity in NIH 3T3 (**E**) and NM18 cells (**F**). For NIH3T3, the results of two controls (control 1 and 2) and 4 KO clones (KO1A, KO1B, KO2A, KO2B) are shown, and for NM18, the results of wild-type (WT) and two LRP1 knockout pools (KO1 and KO2) are shown. Fig. **S4C-D,** **S4E,** and **S4F** correspond to the extended data set for Fig. **4C**, **4D**, and **4E**, respectively.


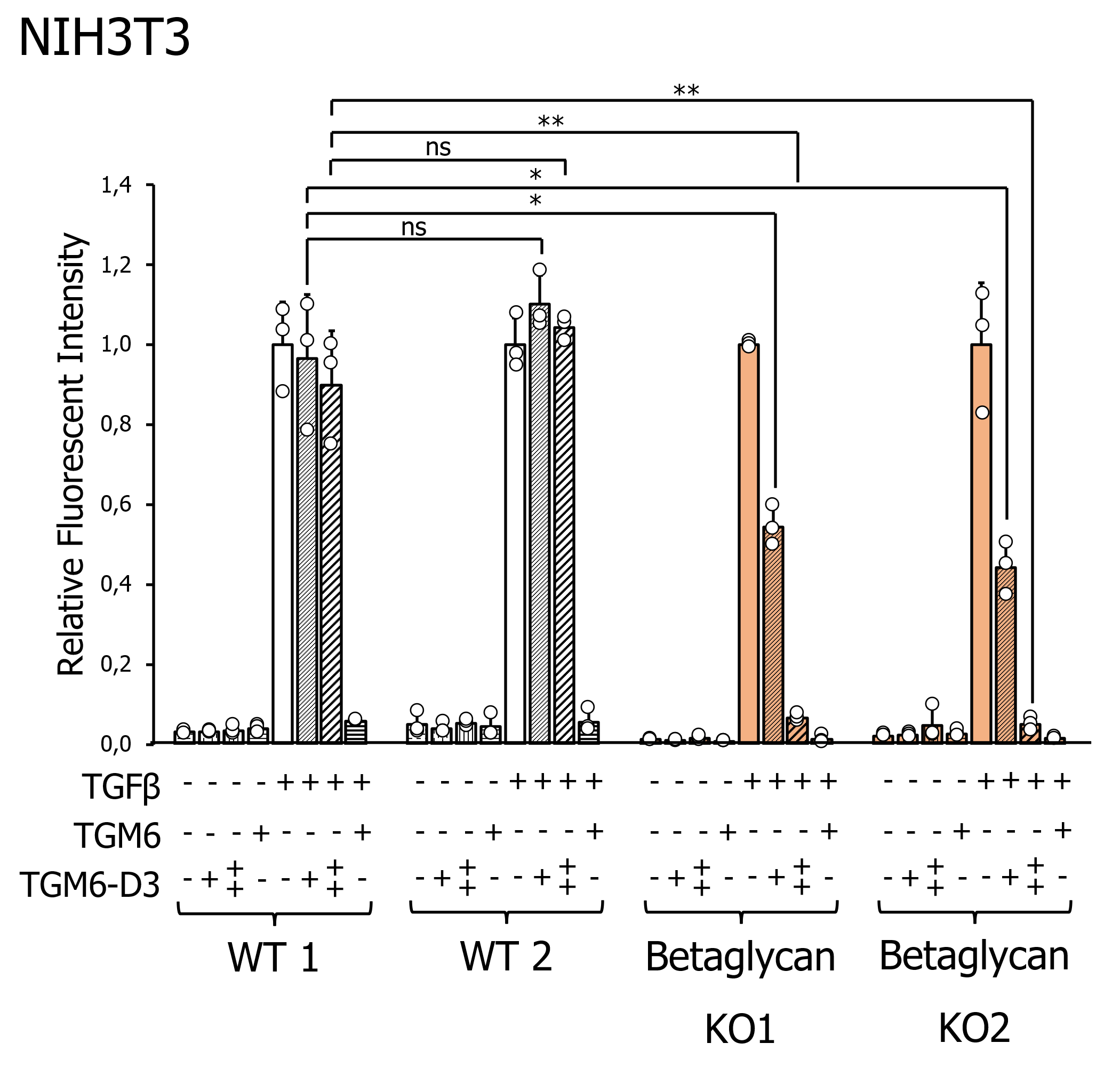


**Figure S5. Effect of betaglycan deficiency on the ability of TGM6-D3 to antagonize TGFβ signaling.** Effect of betaglycan deficiency on the ability of TGM6-D3 to antagonize TGFβ-induced CAGA-dynGFP reporter activity in NIH3T3-CAGA-dynGFP wild-type (WT) clones and betaglycan NIH3T3 KO clones. Cells were pre-incubated with TGM6 (100 ng/ml) or TGM6-D3 (1000 or 5000 ng/ml) for 30 minutes before stimulation with 1 ng/ml TGFβ for 21 hours. The results on two wild-type clones (WT1 and WT2) and two betaglycan KO clones (KO1 and KO2) are shown. KO1 and KO2 were made using different guide RNAs. **S5** corresponds to the extended dataset for Figure **5H**.


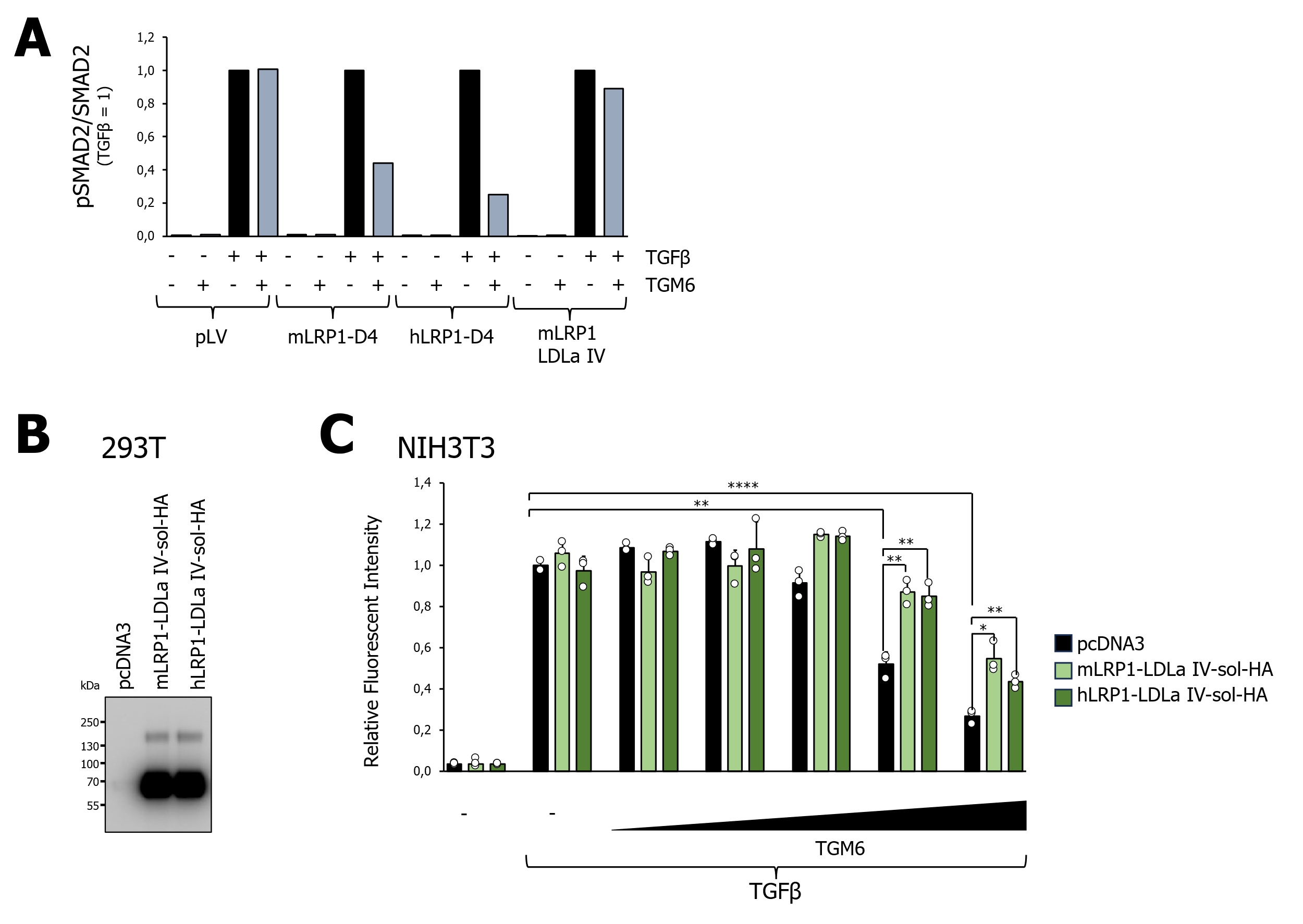


**Figure S6. Effect of soluble LRP1-LDLaIV on TGFβ/SMAD signaling. (A)** Effect of hLRP1-D, mLRP1-D4, and mLRP1 LDLaIV on TGM6-induced inhibition of TGFβ signaling in LRP1 KO cells as measured by SMAD2 phosphorylation. pSMAD2/SMAD2 ratios of Western blot results of Figure **6D** are plotted in the graph. (**B**) Expression analysis of soluble mLRP1-LDLaIV or hLRP1-LDLaIV by western blot analysis of conditioned media of transfected HEK293T cells. (**C**) Effect of mLRP1-LDLaIV or hLRP1-LDLaIV on TGM6-mediated antagonism of TGFβ/SMAD3-induced transcriptional response in NM18 cells. TGM6 (2,5, 10, 25, 50, or 100 ng/ml) was pre-incubated with conditioned media containing either soluble mLRP1-LDLaIV or soluble hLRP1-LDLaIV. Subsequently, this was used to pre-treat NM18 cells (30 minutes) before stimulation with 1 ng/ml TGFβ for 21 hours.


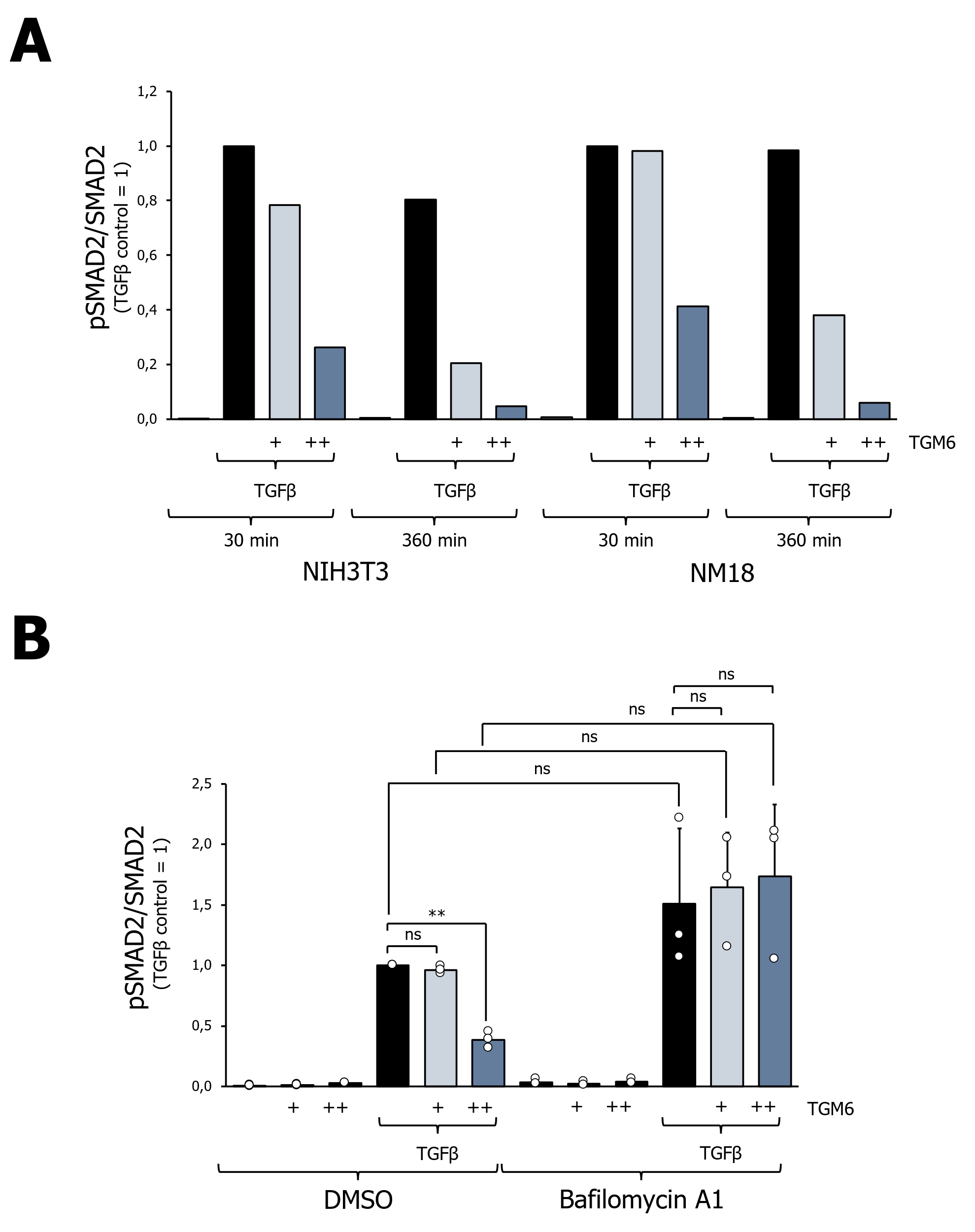


**Figure S7. TGM6 antagonizes TGFβ signaling by inducing LRP1-dependent TGFBR2 lysosomal degradation. A** corresponds to the quantification of the pSMAD2/SMAD2 ratios of the results shown in Figure **7C**, and **B** corresponds to the quantification of pSMAD2/SMAD2 ratios of which the result of one experiment is shown in Figure **7F**.


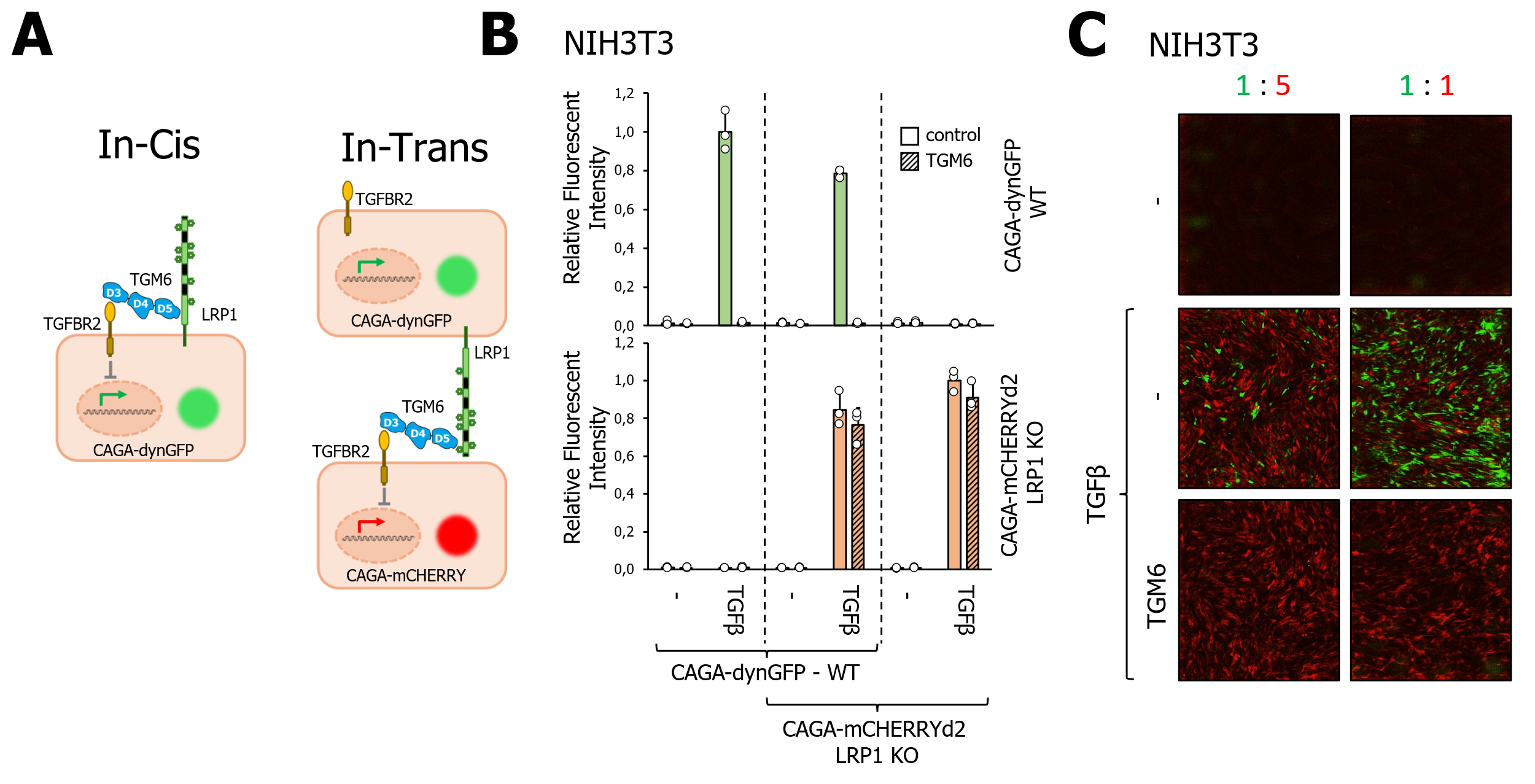


**Figure S8. TGM6 elicits cellular effects in-*cis* and not in-*trans***. (**A**) Schematic representation of TGM6 signaling in-*cis* or in-*trans*. (**B** and **C**) Effect of TGM6 on NIH3T3-CAGA-dynGFP cells (TGFBR2+LRP1+) and NIH3T3-CAGA-mCHERRYd2 cells deficient in LRP1 (TGFBR2+LRP1-), either as mono- or mixed cultures in two different ratios. Cells were challenged with 100 ng/ml TGM6 and/or 1 ng/ml TGFβ, and the CAGA transcriptional response in homogeneous or mixed-culture cells was measured after 21 hours. Quantification is shown in **B**, and representative fluorescent images are shown in **C**.


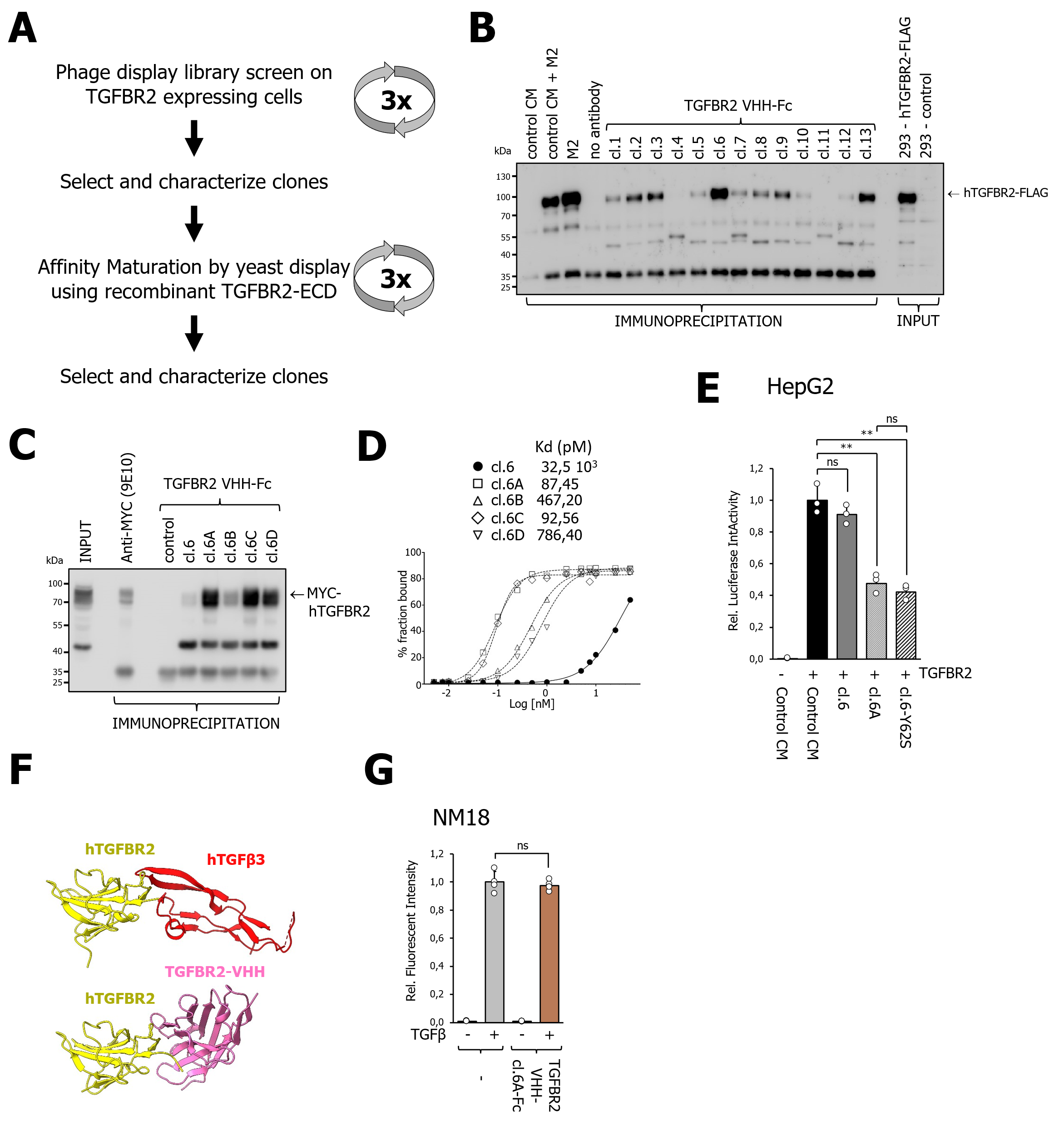


**Figure S9. Generation and characterization of TGFBR2 VHH. (A)** Schematic of the experimental flow that was followed for the identification of a VHH nanobody recognizing TGFBR2 ECD with high affinity. (**B** and **C**) Analysis of the immunoprecipitation efficiency of TGFBR2 VHH clones isolated by (**B**) phage display library screen using TGFBR2 overexpressing HEK 293T cells and (**C**) affinity maturation by yeast display using recombinant TGFBR2 ECD. (**D**) Apparent affinity (Kd) measurements of various TGFBR2-VHH clones on TGFBR2 ECD. (**E**) Effect of various TGFBR2 VHH clones on CAGA-luciferase transcriptional reporter response driven by ectopic expression of TGFBR2 (in the absence of exogenous TGFβ). (**F**) Comparison of the crystal structure of hTGFβ3 to hTGFBR2 ECD (top panel) (PDB 1KTZ [13]) to the AlphaFold prediction of hTGFBR2 ECD in complex with TGFBR2-VHH-cl.6-Y62S. hTGFBR2 is in yellow, hTGFβ3 is in red, and TGFBR2-VHH is in magenta. (**G**) Lack of effect of TGFBR-VHH cl.6 on TGFβ/SMAD3-induced transcriptional response (using CAGA-eGFP transcriptional reporter) in NM18 cells stimulated with TGFβ (1 ng/ml).


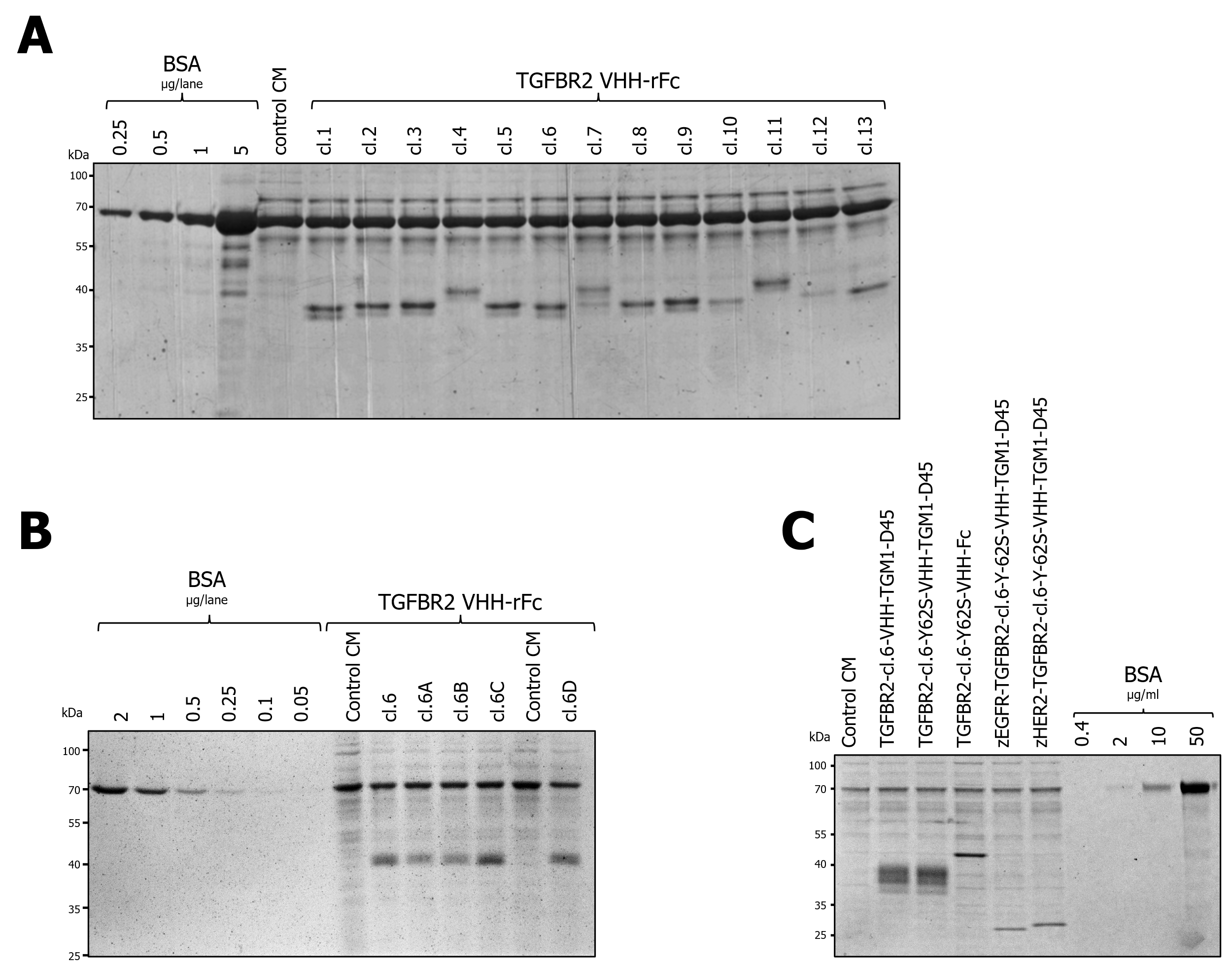


F**igure S10. Expression controls the TGFBR2 VHH fusion proteins.** (**A**, **B,** and **C**) Expression and purity controls of the various TGFBR2-based fusion proteins used in Fig. **S9** and **9**. Proteins were separated by SDS-PAGE and visualized by Coomassie staining. Different amounts of bovine serum albumin (BSA) were used to estimate protein concentrations of TGFBR VHH fusion proteins. CM, conditioned medium.

**
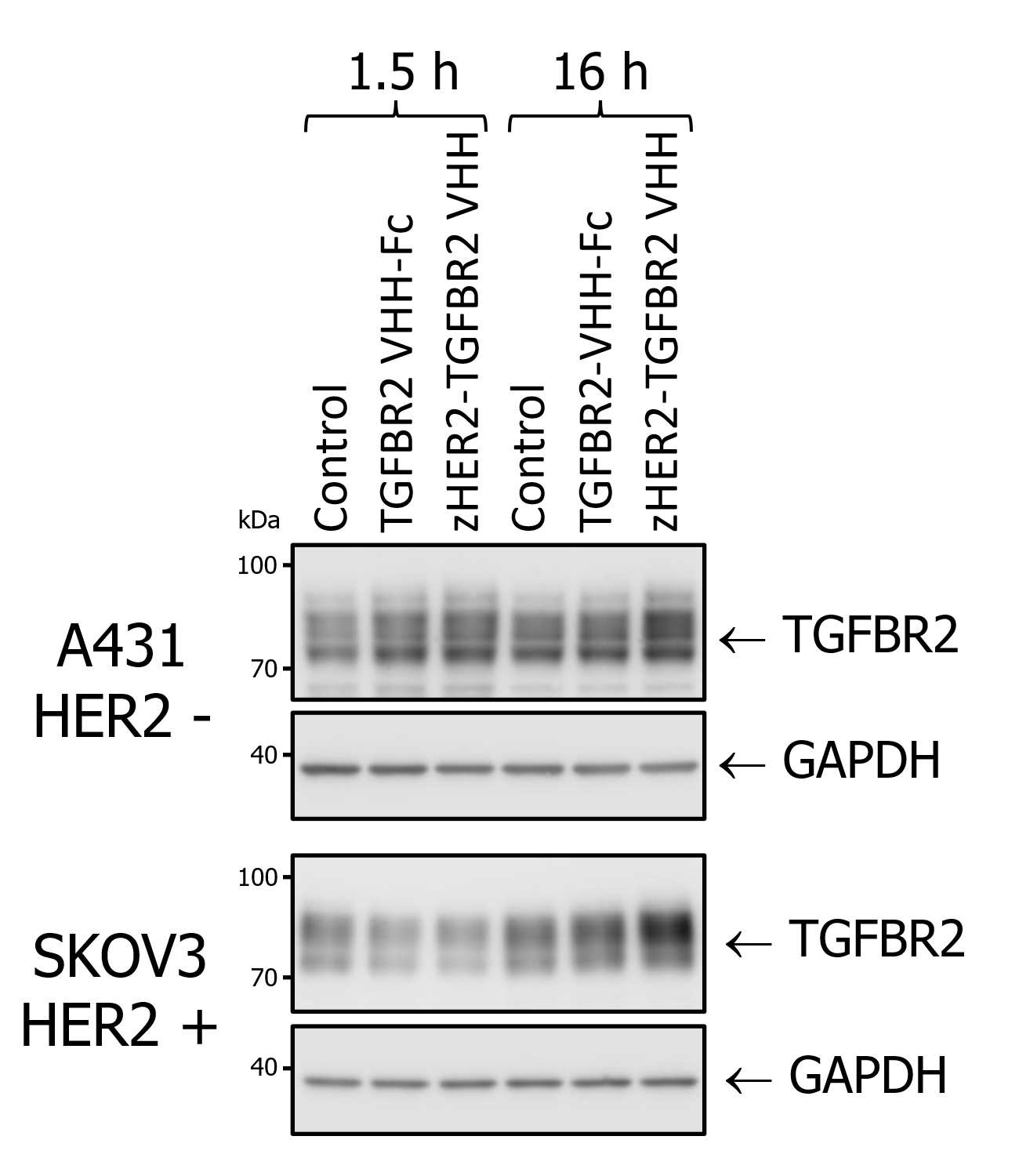
**

**Figure S11. Lack of effect of zHER2-TGFBR2 VHH on TGFBR2 expression.** Western blot analysis of TGFBR2 expression in A431 (expressing no endogenous HER2) and SKOV3 cells (expressing high levels of endogenous HER2) after treatment with TGFBR2 VHH Fc or zHER2-TGFBR2 VHH. Treatment was performed for 1.5 hours or 16 hours, corresponding to the assay times for pSMAD2 and transcriptional response assays, respectively.

Table S1.

**X-ray data collection, reduction, and refinement of the TGM6-D3:mTGFBR2 complex**

| **Supplementary Table 1. X-ray data collection, reduction, and refinement of the TGM6-D3:mTGFBR2 complex** | |
| --- | --- |
|  |  |
| **Data Collection** |  |
| X-Ray Source | NSLS-II BEAMLINE 17-ID-1 |
| Wavelength (Å) | 0.92010 A |
| Detector | DECTRIS EIGER X 9M |
| Number of Crystals | 1 |
| PDB Deposition | 9YJ4 |
|  |  |
| **Data Reduction^a^** |  |
| Space Group | P2_1_2_1_2_1_ |
| a, b, c (Å) | 53.35, 92.59, 93.39 |
| α. β, γ (°) | 90, 90, 90 |
| Completeness (%) | 92.2 (42.0) |
| Resolution (Å) | 32.876 (2.517) |
| R_meas_ | 0.257 (2.171) |
| R_pim_ | 0.089 (0.659) |
| <I/σ(I)> | 6.5 (1.4) |
| Redundancy or Multiplicity | 8.0 (10.5) |
| CC_1/2_ | 0.991 (0.369) |
| CC* | 0.998 (0.734) |
| Proteins/ASU | 2 |
| Percent Solvent (%) | 54.7 |
| Matthews Coefficient | 2.71 |
| Wilson B-Factor (Å2) | 56.96 |
| No. Observations | 100418 (6616) |
| No. Reflections | 12574 (629) |
|  |  |
| **Refinement** |  |
| Resolution (Å) | 32.898 - 2.517 |
| R_Work_ / R_Free_ | 0.2585 / 0.2880 |
| R.M.S. Deviations |  |
| Bond-Lengths (Å) | 0.0122 |
| Bond-Angles (°) | 1.6554 |
| Residues | 378 |
| Atoms / Non-Hydrogens | 3150 / 3150 |
| Protein: Overall / Heavy / Backbone | 3002 / 3002 / 1512 |
| Ions and Ligands: Overall / Heavy | 11 / 11 |
| Waters | 137 |
| B-factors | 64.15 |
| Protein: Overall / Heavy / Backbone | 64.51 / 64.51 / 63.06 |
| Ions and Ligands: Overall / Heavy | 54.05 / 54.05 |
| Waters | 56.91 |
| MolProbity |  |
| Clash Score | 0.00 |
| MolProbity Score | 0.80 |
| Ramachandran (Allowed, Favored, Outliers) | 99.73 %, 95.63 %, 0.27 % |
| Rotamers (Allowed, Favored, Outliers, Clashes) | 100%, 100%, 0%, 0% |
| ^a^ The numbers in the Data Reduction section correspond to the value for the overall and in parentheses the high resolution shell | |

Table S2.

**TGFBR2 VHH fusion constructs**

| **Supplementary Table 2.** **TGFBR2 VHH fusion constructs** | | |
| --- | --- | --- |
|  | |  |
| **Construct** | **Sequence** | |
| TGFBR2 VHH cl.6 - TGM1-D4/5 - FLAG (signal peptide) | MYRMQLLSCI ALSLALVTNS ISAMAEVQLQ ASGGGFVQPG GSLRLRCAAS GTTFGDSIMG WFRQAPGKER EFVSAISSRT NIGIYYADSV KGRFAISRDN SKNTVYLQMN SLRAEDTATY YCAMRFEGFK VMYWGQGTQV TVSSAAARCK PLEANESVHY EYFTMTNETD KKKGPPAKVG KSGKYPEHTC VKKVCSKWPY TCSTGGPIFG ECIGATWNFT ALMECINARG CSSDDLFDKL GFEKVIVRKG EGSDSYKDDF ARFYATGSKV IAECGGKTVR LECSNGEWHE PGTKTVHRCT KDGIRTLLEA ARGGPDYKDD DDK | |
| TGFBR2 VHH cl.6-Y62S - TGM1-D4/5 - FLAG (signal peptide) | MYRMQLLSCI ALSLALVTNS ISAMAEVQLQ ASGGGFVQPG GSLRLRCAAS GTTFGDSIMG WFRQAPGKER EFVSAISSRT NIGISYADSV KGRFAISRDN SKNTVYLQMN SLRAEDTATY YCAMRFEGFK VMYWGQGTQV TVSSAAARCK PLEANESVHY EYFTMTNETD KKKGPPAKVG KSGKYPEHTC VKKVCSKWPY TCSTGGPIFG ECIGATWNFT ALMECINARG CSSDDLFDKL GFEKVIVRKG EGSDSYKDDF ARFYATGSKV IAECGGKTVR LECSNGEWHE PGTKTVHRCT KDGIRTLLEA ARGGPDYKDD DDK | |
| TGFBR2 VHH cl.6-Y62S - rFc (signal peptide) | MYRMQLLSCI ALSLALVTNS ISAMAEVQLQ ASGGGFVQPG GSLRLSCAAS GTTFGDSIMG WFRQAPGKER EFVSAISSRT NIGISYADSV KGRFTISRDN SKNTVYLQMN SLRAEDTATY YCAMRFEGFK VMYWGQGTQV TVSSAAARSS KPTCPPPELL GGPSVFIFPP KPKDTLMISR TPEVTCVVVD VSQDDPEVQF TWYINNEQVR TARPPLREQQ FNSTIRVVST LPIAHQDWLR GKEFKCKVHN KALPAPIEKT ISKARGQPLE PKVYTMGPPR EELSSRSVSL TCMINGFYPS DISVEWEKNG KAEDNYKTTP AVLDSDGSYF LYSKLSVPTS EWQRGDVFTC SVMHEALHNH YTQKSISRSP GK | |
| zEGFR - linker - TGFBR2 VHH cl.6-Y62S -10xHIS (signal peptide) | MYRMQLLSCI ALSLALVTNS MARVDNKFNK EMWAAWEEIR NLPNLNGWQM TAFIASLVDD PSQSANLLAE AKKLNDAQAP KGGGGSGGGG SGGGGSSMAE VQLQASGGGF VQPGGSLRLS CAASGTTFGD SIMGWFRQAP GKEREFVSAI SSRTNIGISY ADSVKGRFTI SRDNSKNTVY LQMNSLRAED TATYYCAMRF EGFKVMYWGQ GTQVTVSSAA AHHHHHHHHH H | |
| zHER2 - linker - TGFBR2 VHH cl.6-Y62S - 10xHIS (signal peptide) | MYRMQLLSCI ALSLALVTNS MARVDNKFNK EMRNAYWEIA LLPNLNNQQK RAFIRSLYDD PSQSANLLAE AKKLNDAQAP KGGGGSGGGG SGGGGSSMAE VQLQASGGGF VQPGGSLRLS CAASGTTFGD SIMGWFRQAP GKEREFVSAI SSRTNIGISY ADSVKGRFTI SRDNSKNTVY LQMNSLRAED TATYYCAMRF EGFKVMYWGQ GTQVTVSSAA AHHHHHHHHH H | |

**Table S3.**

**TGM6 fusion constructs**

| **Supplementary Table 3. TGM6 fusion constructs** | |
| --- | --- |
|  |  |
| **Construct** | **Sequence** |
| TGM6-D3/TGM1-D45  (Signal Peptide) | METDTLLLWV LLLWVPGSTG DAAQPAGSSC PPLPDDETVW YEYYGYVDGR HTVGDAAIKD SLENYPPNTH ARRHCKALSK KADPGEFVAI CYQRRGTSES QWQYYPRIAS CPDPRCKPLE ANESVHYEYF TMTNETDKKK GPPAKVGKSG KYPEHTCVKK VCSKWPYTCS TGGPIFGECI GATWNFTALM ECINARGCSS DDLFDKLGFE KVIVRKGEGS DSYKDDFARF YATGSKVIAE CGGKTVRLEC SNGEWHEPGT KTVHRCTKDG IRTLAAARGG PEQKLISEED LNSAVDHHHH HH |
| TGM1-D12/TGM6-D345  (Signal Peptide) | METDTLLLWV LLLWVPGSTG DAAQPAGCMP FSDEAATYKY VAKGPKNIEI PAQIDNSGMY PDYTHVKRFC KGLHGEDTTG WFVGICLASQ WYYYEGVQEC DDRRCSPLPT NDTVSFEYLK ATVNPGIIFN ITVHPDASGK YPELTYIKRI CKNFPTDSNV QGHIIGMCYN AEWQFSSTPT CPASSCPPLP DDETVWYEYY GYVDGRHTVG DAAIKDSLEN YPPNTHARRH CKALSKKADP GEFVAICYQR RGTSESQWQY YPRIASCPDP RCKPLEKNDS VSYEYFTKPT KGLKMGSITK PDKSGKYPEE TFVRRYCNDL PRNSLAQGKT YAECLDSEWK LKNLPDCRFA AGCDEEYLLE KLMFVDISYW GKDAAKFSDD KTYRYYRPGS KVTAKCKGKS VKLTCVDGGY WVTVDGRKAL CTAAARGGPE QKLISEEDLN SAVDHHHHHH |
| zHER2-linker-TGM6-D3  (STREP II tag) | MAAQPAVDNK FNKEMRNAYW EIALLPNLNN QQKRAFIRSL YDDPSQSANL LAEAKKLNDA QAPKGGGGSG GGGSGGGGSS MACSSCPPLP DDETVWYEYY GYVDGRHTVG DAAIKDSLEN YPPNTHARRH CKALSKKADP GEFVAICYQR RGTSESQWQY YPRIASCPDP AAAWSHPQFE K |

**Table S4.**

**TGM6-D3 and TGFBR2 constructs used in this study**

| **Supplementary Table 4. TGM6-D3 and TGFBR2 constructs used in this study** |
| --- |

|  |  |  |
| --- | --- | --- |
| **Construct** | **Residue range and features*** | **Sequence** |
| TGM6-D3 | Residues 15-102 of *H. polygyrus* TGF-β Mimic 6  (NCBI MG429741)  Expressed as a Thioredoxin- His10-Linker-Thrombin-Linker-TGM6-D3 fusion | MSDKIIHLTD DSFDTDVLKA DGAILVDFWA EWCGPCKMIA PILDEIADEY QGKLTVAKLN IDQNPGTAPK YGIRGIPTLL LFKNGEVAAT KVGALSKGQL KEFLDANLAG SGSGHMSSGH HHHHHHHHHS SGGSGLVPRG SGTGSSCPPL PDDETVWYEY YGYVDGRHTV GDAAIKDSLE NYPPNTHARR HCKALSKKAD PGEFVAICYQ RRGTSESQWQ YYPRIASCPD P |
| hTGFBR2 | Residues 38-159 of the human TGF-β type II receptor (NCBI NP_003233)  Expressed as TβRII alone | MVTDNNGAVK FPQLCKFCDV RFSTCDNQKS CMSNCSITSI CEKPQEVCVA VWRKNDENIT LETVCHDPKL PYHDFILEDA ASPKCIMKEK KKPGETFFMC SCSSDECNDN IIFSEEYNTS  NPD |
| hTGFBR2 F47L | Residues 38-159 of the human TGF-β type II receptor (NCBI NP_003233)  Expressed as TβRII alone | MVTDNNGAVK LPQLCKFCDV RFSTCDNQKS CMSNCSITSI CEKPQEVCVA VWRKNDENIT LETVCHDPKL PYHDFILEDA ASPKCIMKEK KKPGETFFMC SCSSDECNDN IIFSEEYNTS  NPD |
| hTGFBR2 F47L, S75A, D141E | Residues 38-159 of the human TGF-β type II receptor (NCBI NP_003233)  Expressed as TβRII alone | MVTDNNGAVK LPQLCKFCDV RFSTCDNQKS CMSNCSITAI CEKPQEVCVA VWRKNDENIT LETVCHDPKL PYHDFILEDA ASPKCIMKEK KKPGETFFMC SCSSEECNDN IIFSEEYNTS  NPD |
| mTGFBR2 | Residues 38-159 of the mouse TGF-β type II receptor (NCBI NP_083851)  Expressed as TβRII alone | MASDNGGAVK LPQLCKFCDV RLSTCDNQKS CMSNCSITAI  CEKPHEVCVA VWRKNDKNIT LETVCHDPKL TYHGFTLEDA  ASPKCVMKEK KRAGETFFMC ACNMEECNDY IIFSEEYTTS  SPD |
| mTGFBR2-L47F | Residues 38-159 of the mouse TGF-β type II receptor (NCBI NP_083851)  Expressed as TβRII alone | MASDNGGAVK FPQLCKFCDV RLSTCDNQKS CMSNCSITAI  CEKPHEVCVA VWRKNDKNIT LETVCHDPKL TYHGFTLEDA  ASPKCVMKEK KRAGETFFMC ACNMEECNDY IIFSEEYTTS  SPD |
| mTGFBR2-L47F, A75S, E141D | Residues 38-159 of the mouse TGF-β type II receptor (NCBI NP_083851)  Expressed as TβRII alone | MASDNGGAVK FPQLCKFCDV RLSTCDNQKS CMSNCSITSI  CEKPHEVCVA VWRKNDKNIT LETVCHDPKL TYHGFTLEDA  ASPKCVMKEK KRAGETFFMC ACNMDECNDY IIFSEEYTTS  SPD |
| *All residue numbering begins with the N-terminal methionine of the naturally occurring signal peptide | | |

**Table S5.**

**Target sequences guide RNA’s**

| **Supplementary Table 5. Target sequences guide RNA’s** | |
| --- | --- |
|  |  |
| **Target** | **sequence** |
| Mouse Lrp1 – guide 1 | CGTGGACCAGACTCGCCCAGGGG |
| Mouse Lrp1 – guide 2 | ACTGCTTGGAGATACACCGGTGG |
| Mouse Betaglycan – guide 1 | CGCTCATCAGGACAAACACAGGG |
| Mouse Betaglycan – guide 2 | TGTCCTGATGAGCGCCTGCCTGG |
| Mouse Tgfbr2 – guide 1 | ACCTGCAGGAGTACCTCACGAGG |
| Mouse Tgfbr2 – guide 2 | GATCACGGCGTTCCACGCGAAGG |

**Table S6.**

**Antibodies**

| **Supplementary Table 6. Antibodies** | | |
| --- | --- | --- |
|  |  |  |
| **Antibody** | **Company** | **Catalog number or Reference** |
| pSMAD2 |  | Persson et. al. |
| SMAD2 | Cell Signaling Technologies | 3102 |
| TGFBR1 (ALK5) | Abcam | Ab235578 |
| TGFBR2 | Abcam | Ab259360 |
| TGFBR3 (Betaglycan) | Cell Signaling Technologies | 5544 |
| LRP1 alpha | Thermo Scientific | L2420 |
| LRP1 beta | Cell Signaling Technologies | 64099 |
| E-cadherin | Becton Dickinson | 610181 |
| N-cadherin | Becton Dickinson | 610920 |
| GAPDH | Millipore | MAB374 |
| HER2 | Abcam | Ab134182 |
| MYC (9E10) | Santa Cruz | SC-40 |
| HA (12CA5) | Roche | 11583816001 |
| FLAG-M2 | Sigma | F3165 |
